# Comparison of effectiveness and safety of bexagliflozin and other sodium-glucose cotransporter 2 inhibitors for type 2 diabetes mellitus in adults: systematic review and network meta-analysis of randomized controlled trials

**DOI:** 10.3389/fendo.2026.1843370

**Published:** 2026-05-13

**Authors:** Shan Gao, Fuyong Zhang, Xingxing Xie, Ling Fan

**Affiliations:** 1 Department of Pharmacy, West China School of Medicine, Sichuan University, Sichuan University affiliated Chengdu Second People’s Hospital, Chengdu Second People’s Hospital, Chengdu, Sichuan, China; 2 Department of Pharmacy, Deyang People’s Hospital, Deyang, Sichuan, China; 3 Department of Pharmacy, Yaan People’s Hospital, Yaan, Sichuan, China; 4 Department of Good Clinical Practice, Yaan People’s Hospital, Yaan, Sichuan, China

**Keywords:** bexagliflozin, network meta-analysis, SGLT2 inhibitors, systematic review, type 2 diabetes mellitus

## Abstract

**Background:**

Bexagliflozin exerts definite efficacy in the treatment of type 2 diabetes mellitus (T2DM). However, whether this novel sodium-glucose cotransporter 2 (SGLT2) inhibitor is superior to other SGLT2 inhibitors remains to be elucidated. We therefore performed this network meta-analysis (NMA) to compare bexagliflozin with other SGLT2 inhibitors and establish an efficacy hierarchy in T2DM management.

**Methods:**

We systematically searched PubMed, Embase, Web of Science and the ClinicalTrials.gov registry for eligible randomized controlled trials (RCTs) published up to January 2026. Statistical analysis was conducted using Stata 14.0. Risk of bias was assessed by the Cochrane tool, evidence certainty was evaluated using the Confidence in Network Meta-Analysis (CINeMA) approach, and intervention ranking was performed using surface under the cumulative ranking curve (SUCRA) values.

**Results:**

This NMA included 48 studies with 26,838 patients. Bexagliflozin significantly reduced HbA1c, fasting plasma glucose (FPG), body weight, systolic blood pressure (SBP) and diastolic blood pressure (DBP) compared with placebo. For HbA1c reduction, canagliflozin (300 mg, 100 mg) and empagliflozin 25 mg were more effective than bexagliflozin, while bexagliflozin was comparable to other SGLT2 inhibitors. For FPG reduction, canagliflozin 300 mg and empagliflozin 25 mg showed slightly greater effects than bexagliflozin, with no significant differences between bexagliflozin and other comparators. Bexagliflozin was superior to dapagliflozin 5 mg but slightly inferior to canagliflozin 300 mg for weight loss, while showing comparable efficacy to other SGLT2 inhibitors. It achieved similar SBP and DBP reduction to other SGLT2 inhibitors, with a significantly greater DBP-lowering effect than empagliflozin 10 mg. Bexagliflozin had a lower incidence of urinary tract infection than dapagliflozin (5 mg, 10 mg), with comparable safety to other agents and placebo. Canagliflozin 300 mg showed the best efficacy for HbA1c, FPG and weight control.

**Conclusion:**

Bexagliflozin demonstrates comparable efficacy to most SGLT2 inhibitors in T2DM patients, with a relatively prominent benefit in body weight reduction and a similar safety profile. Canagliflozin 300 mg provides more effective glycemic and weight control.

## 1 Introduction

The International Diabetes Federation (IDF) reported in the latest atlas data that the global prevalence of diabetes among adults reached an alarming 11.11% in 2024, with approximately 589 million adults living with the condition—equating to roughly one in nine adults affected by diabetes worldwide. Of these cases, an estimated 252 million individuals remain undiagnosed, placing them at elevated risk of diabetes-related complications and premature mortality. Additionally, one in eight adults is considered to be at high risk of developing type 2 diabetes. If current epidemiological trends persist unabated, the global adult diabetes prevalence is projected to rise to 12.96% by 2050, with the total number of affected individuals increasing to 853 million (1–3). At the pathological level, insufficient insulin secretion or insulin resistance results in a persistent hyperglycemic state in T2DM patients, leading to chronic impairment and functional decline of blood vessels, nerves, the brain, and other associated tissues and organs (4, 5). Accordingly, the core goal of T2DM management is to efficiently control blood glucose levels and reduce the likelihood of macrovascular as well as microvascular complications (6).

Sodium-glucose cotransporter 2 inhibitors represent a class of glucose-lowering agents that exert their therapeutic effects by suppressing glucose reabsorption in the proximal renal tubule, thereby promoting urinary glucose excretion and lowering serum glucose concentrations (7). Notably, these agents exhibit favorable therapeutic efficacy in optimizing glycemic control and lowering body weight among individuals with T2DM (8). In clinical practice, SGLT2 inhibitors are frequently co-administered with metformin. The combination of SGLT2 inhibitors and metformin has been endorsed as a dual first-line therapeutic strategy for T2DM in global clinical guidelines, attributed to its robust efficacy in sustaining glycemic control and versatile metabolic benefits (9). Therefore, over the past years, several SGLT2 inhibitors have been approved by the Food and Drug Administration (FDA) (dapagliflozin, empagliflozin, canagliflozin, ertugliflozin and bexagliflozin).

Bexagliflozin is a novel oral SGLT2 inhibitor approved by the FDA in 2023 for the management of T2DM. Earlier meta-analyses have shown that bexagliflozin at a dose of 20 mg substantially lowers HbA1c, FPG (glycemic effects), body weight, and SBP (extra-glycemic effects) relative to placebo in patients with T2DM. Regarding safety, bexagliflozin was comparable to the placebo group, and the most frequently reported non-serious adverse effects were urinary tract infection, polyuria, upper respiratory tract infection or nasopharyngitis, diarrhea, hypoglycemia and nausea (10). While these medications have exhibited reliable efficacy in adults with T2DM across several clinical trials, head-to-head randomized controlled trials comparing bexagliflozin with other clinically approved SGLT2 inhibitors are still insufficient. As a result, their comparative effectiveness and safety have not been fully established.

Through the integration of more extensive studies, network meta-analysis facilitates indirect comparisons between therapeutic interventions for which direct comparative data is limited. Through synthesizing diverse research studies, NMA enhances statistical power and analytical accuracy. Beyond visualizing the evidence network via network diagrams—thereby identifying research gaps—NMA also ranks interventions according to predefined endpoints. Consequently, the core objective of this research was to evaluate the comparative effectiveness and safety of bexagliflozin against other available SGLT2 inhibitors in T2DM treatment via NMA.

## 2 Methods

This NMA was carried out in adherence to the Preferred Reporting Items for Systematic Reviews and Meta-Analyses (PRISMA) reporting guidelines (11). Our protocol has been registered in PROSPERO with the registration number CRD420251025292 (available at https://www.crd.york.ac.uk/PROSPERO/view/CRD420251025292). Details summarizing the checklist are documented in **Supplementary Table 1**.

### 2.1 Search strategy

The literature retrieval encompassed four prominent electronic databases, namely PubMed, Embase, Web of Science, and the ClinicalTrials.gov registry (https://www.clinicaltrials.gov/). The literature search was conducted from the inception of each database through January 2026, without any restrictions on the language of publication. Detailed database-specific search strategies are comprehensively documented in **Supplementary Table 2**.

### 2.2 Study selection and eligibility criteria

Two reviewers (S. Gao and L. Fan) independently screened all included studies. Initially, literature was filtered by reviewing titles and abstracts, followed by full-text retrieval and assessment in accordance with the predefined inclusion criteria. Disagreements arising between the authors were resolved by a third investigator (X. Xie). The eligibility of included studies was determined in accordance with all subsequent inclusion criteria included:

1. RCTs recruited adult patients with T2DM in accordance with established international diagnostic guidelines.
2. RCTs utilizing SGLT2 inhibitors(dapagliflozin, empagliflozin, canagliflozin, ertugliflozin, bexagliflozin).
3. RCTs assessing placebo, either used as monotherapy or combined with other therapeutic approaches.
4. Studies were required to report at least the following outcome measures: reductions in HbA1c, FPG, body weight loss, as well as changes in SBP and DBP. For safety outcomes, the proportion of patients experiencing hypoglycemia, urinary tract infections, and genital mycotic infection was documented.

Exclusion criteria: (1) animal studies; (2) letters, reviews, meta-analyses, and commentaries; (3) studies lacking data required for efficacy and safety assessments; (4) studies lasting < 12 weeks.

### 2.3 Data extraction

Two authors (S. Gao and L. Fan) independently extracted data using a standardized data collection form, with the extracted information subsequently verified by a third author (F. Zhang). The following data points were retrieved: main authors, year of publication, NCT number, number of participants, participants’ age, gender, baseline body weight, follow-up duration, dosing regimen for intervention, sample size, outcome measures, and HbA1c level.

### 2.4 Quality assessment

Two independent reviewers performed the methodological quality assessment for each included study using the Cochrane Collaboration’s Risk of Bias Tool (12). Based on the results of this appraisal, all included studies were stratified into three bias risk categories: low risk, high risk, and unclear risk. Discrepancies emerging during the assessment process were addressed via structured deliberation among the research team. Review Manager 5.4 was applied to perform data processing and present results.

### 2.5 Certainty assessment

The Confidence in Network Meta-Analysis tool was applied to appraise the credibility of all pairwise comparisons (13), which encompasses six key assessment domains: within-study bias, indirectness, reporting bias, imprecision, inconsistency, and heterogeneity. Based on the severity of bias identified in each domain, we assigned one of three ratings: no concern (no downgrading applied), some concern (one-level downgrade), or major concern (two-level downgrade). We then synthesized the ratings across all domains to obtain an overall confidence level for each comparison, with levels stratified as very low, low, moderate, or high. Additionally, a comprehensive record of all reasons for downgrading was compiled for every individual comparison analyzed.

### 2.6 Statistical analysis

We performed a frequentist network meta-analysis using aggregate data in Stata 14.0 (StataCorp, TX, USA). The main goal was to compare the efficacy and safety of different SGLT2 inhibitors. Network meta-analysis is a statistical method that allows simultaneous comparison of three or more interventions. It combines both direct and indirect evidence from a connected study network (14). This method can provide effect estimates for all pairwise comparisons in the network. These estimates are usually more precise than single direct or indirect comparisons (15). In this study, we used frequentist random-effects NMA models. For continuous outcomes, we calculated mean differences (MD). These outcomes included changes in HbA1c, FPG, body weight, SBP, and DBP. For dichotomous outcomes, we estimated odds ratios (OR) and 95% confidence intervals (CI). We assumed a common between-study heterogeneity variance across all comparisons. Heterogeneity was assessed through two complementary approaches. First, we evaluated the common between-study variance (τ^2^) for each outcome and compared it with previous empirical distributions (16, 17). Second, we used the I^2^ statistic to measure heterogeneity. An I^2^ value ≤ 50% was considered low heterogeneity. We also evaluated inconsistency in the network meta-analysis at both global and local levels. We performed a global test to check overall inconsistency, based on the p-value. Local inconsistency refers to differences between direct and indirect estimates. We used the node-splitting method to detect this kind of inconsistency (18, 19). A p-value < 0.05 suggested potential local inconsistency, which may affect the reliability of the results. If inconsistency was found, we further explored the possible reasons. We used funnel plots and Egger’s regression test to evaluate publication bias. A p-value < 0.05 was considered statistically significant. We also conducted sensitivity analyses by excluding studies with high heterogeneity or small sample sizes (n < 50).

## 3 Results

### 3.1 Study selection and characteristics

**Figure 1:**
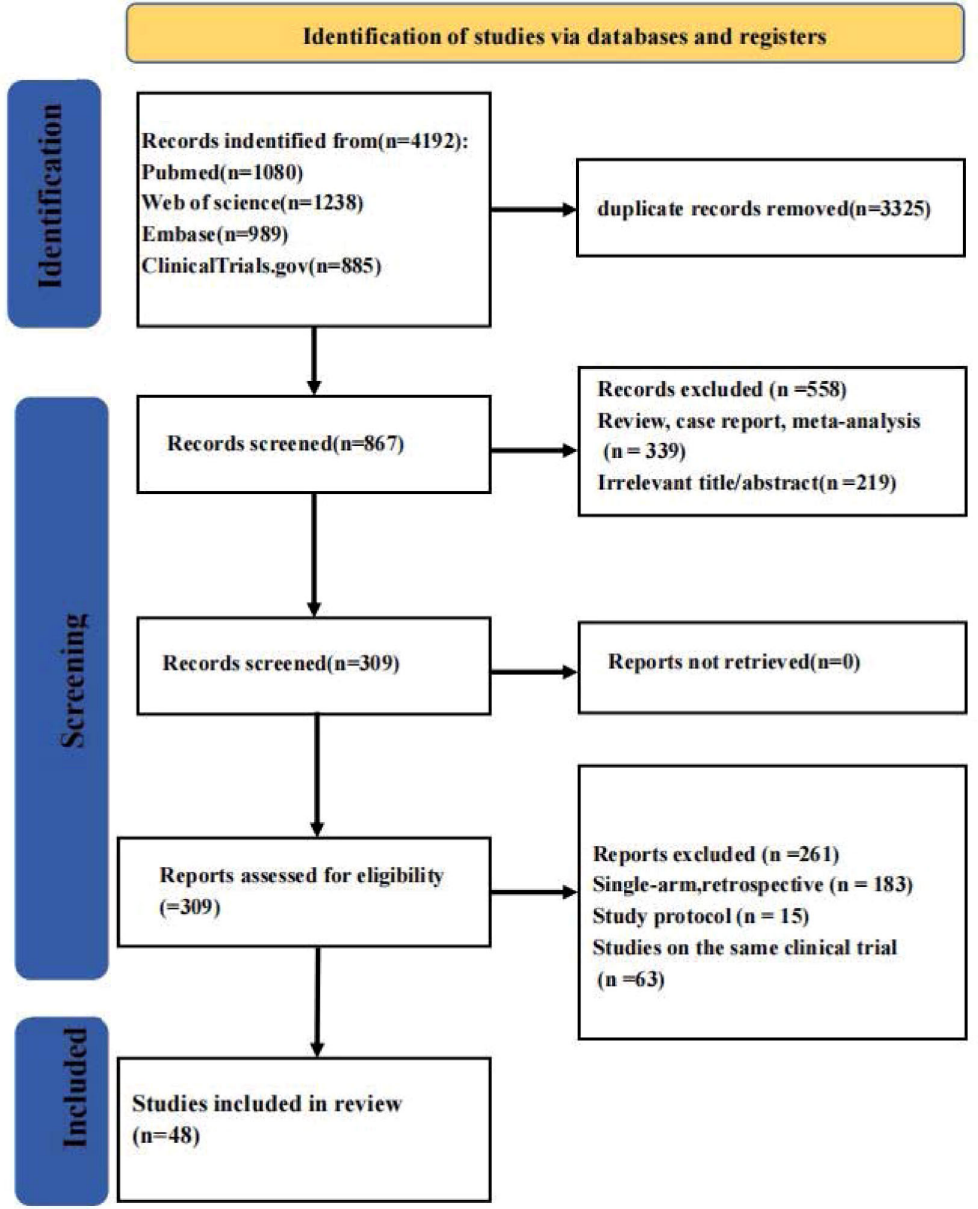
Flow diagram of the study selection process.

We retrieved 4192 records from databases. After excluding 3325 duplicates, 867 records entered the abstract screening stage, with 219 excluded for irrelevance and 339 excluded for their nature as meta-analyses or reviews. The 309 articles that passed this step underwent rigorous eligibility evaluation, from which 261 were excluded on account of three limitations: single-arm or retrospective designs (n=183), study protocols (n=15), and overlapping reports of the same clinical trial (n=63). Ultimately, 48 studies (20–67) met all inclusion criteria and were incorporated into the systematic review, with the entire screening workflow illustrated in **Figure 1**.

Detailed characteristics of all included studies are summarized in **Table 1**. The NMA encompassed a total of 26,838 patients diagnosed with T2DM. The mean sample size per study was 216 (15–2747). The average age of the enrolled patients reached 57.95 years (50.6–69.9). Of the total cohort, 16,668 patients (62.1%) were male. With regard to baseline clinical parameters, the mean HbA1c level stood at 8.04% (7.38–8.89), the mean FPG concentration was 9.02mmol/L (7.63–10.1), and the mean BW was 82.6 kg (65.81–96.2).

**Table 1:** Characteristics of the included studies.

Study	Trial number	Duration of follow‐up	Study arms	No	Age (years)	Gender male,n(%)	Body weight(kg)	HbA1c (%)	FPG (mmol/L)
Halvorsen YC 2019	NCT01377844	96 weeks	Bexagliflozin 20 mg	145	56.2 (10.9)	67 (46.2)	78.4 (17.1)	NA	9.64 (2.46)
			Placebo	138	54.9 (10.3)	49 (35.5)	79.7 (17.4)	NA	9.23 (2.48)
Halvorsen YD 2020	NCT02390050	12 weeks	Bexagliflozin 20 mg	76	59.5 (10.8)	50 (65.8)	78.8 (16.7)	7.73 (0.45)	8.52 (1.44)
			Placebo	72	58.8 (10.4)	42 (58.3)	78.7 (19.7)	7.63 (0.44)	8.69 (1.82)
Allegretti AS 2019	NCT02836873	24 weeks	Bexagliflozin 20 mg	157	69.3 (8.36)	92 (58.6)	82.90 (20.50)	8.01 (0.78)	8.61 (2.52)
			Placebo	155	69.9 (8.29)	104 (67.1)	82.59 (21.19)	7.95 (0.81)	8.63 (2.24)
Lock JP 2016	NCT02715258	24 weeks	Bexagliflozin 20 mg	138	55.8 (10.21)	66 (47.8)	90.5 (20.5)	8.05 (0.82)	9.39 (1.95)
			Placebo	69	54.7 (11.02)	34 (49.3)	84.6 (19.7)	7.97 (0.75)	9.45 (2.07)
Lock JP 2015	NCT02558296	24 weeks	Bexagliflozin 20 mg	1133	64.4 (7.94)	66(47.8)	94.59 (21.87)	8.32 (0.89)	NA
			Placebo	567	64.6 (8.01)	34(49.3)	92.62 (19.99)	8.33(0.94)	NA
Lock JP 2017	NCT03259789	24 weeks	Bexagliflozin 20 mg	158	55.8 (10.62)	100 (63.3)	84.51 (21.43)	NA	NA
			Placebo	159	52.1 (8.59)	94 (59.1)	87.28 (17.75)	NA	NA
Bailey CJ 2012	NA	24 weeks	Dapagliflozin 5mg	68	51.3 (11.51	32 (47.1)	85.4 (19.43	7.9 (1.03)	8.72 (2.31)
			Placebo	68	53.5 (11.08	37 (54.4)	90.0 (17.98	7.8 (1.12)	8.97 (3.19)
Kaku K 2013	NCT00972244	12 weeks	Dapagliflozin 5mg	58	58.0 (9.5)	47 (81.0)	68.92(12.43)	8.05 (0.66)	9.14 (1.31)
			Dapagliflozin 10mg	52	56.5 (11.5)	39 (75.0)	70.35 (17.48)	8.18 (0.69)	9.08 (1.65)
			Placebo	54	58.4 (10.0)	43 (79.6)	68.88 (14.94)	8.12 (0.71)	8.83 (1.73)
Ferrannini E 2010	NCT00528372	12 weeks	Dapagliflozin 5mg	75	52.6(10.9)	31(38.4)	87.6(17.1)	7.86(0.94)	9.01(2.5)
			Dapagliflozin 10mg	64	50.6(9.97)	24(48.4)	94.2(18.7)	8.01(0.96)	9.26(2.30)
			Placebo	70	52.7(10.3)	31(41.3)	88.8(19.0)	7.84(0.87)	8.88(2.34)
Lambers HHJ 2013	NCT00976495	12 weeks	Dapagliflozin 10mg	24	53.7 (9.4)	16 (66.7)	93.2 (18.0)	7.8 (0.62)	8.8 (2.0)
			Placebo	25	58.0 (9.5)	18 (72.0)	96.2 (19.5)	7.4 (0.78)	8.1 (2.4)
List JF 2009	NCT00263276	12 weeks	Dapagliflozin 5mg	58	55(12)	28 (48)	89(17)	8.0 (0.9)	8.5 (2.66)
			Dapagliflozin 10mg	47	54(9)	25 (53)	86(17)	8.0(0.8)	8.22 (2.11)
			Placebo	54	53(11)	30 (56)	89(18)	7.9(0.9)	8.33 (2.55)
Bailey CJ 2010	NCT00528879	24 weeks	Dapagliflozin 5mg	137	54.3 (9.4)	69 (50)	84.9(7.8)	8.17 (0.96)	9.39(2.72)
			Dapagliflozin 10mg	135	52.7 (9.9)	77 (57)	84.7 (16.3)	7.92(0.82)	8.66(2.15)
			Placebo	137	53.7 (10.3)	76 (55)	87.7 (19.2)	8.11(0.96)	9.19(2.57)
Cefalu WT 2015	NCT01031680	24 weeks	Dapagliflozin 10mg	459	62.8 (7.0)	313 (68.2)	92.6 (20.5)	8.08 (0.80)	8.9 (2.6)
			Placebo	455	63.0 (7.7)	311 (68.6)	93.6 (19.5)	8.18 (0.84)	8.8 (2.3)
Kaku K 2014	NCT03050229	24 weeks	Dapagliflozin 5mg	86	58.6 (10.4)	50 (58.1)	65.81 (14.37)	7.50 (0.72)	7.63 (1.35)
			Dapagliflozin 10mg	88	57.5 (9.3)	53 (60.2)	69.70 (13.82)	7.46 (0.61)	7.70 (1.23)
			Placebo	87	60.4 (9.7)	52 (59.8)	65.96 (12.91)	7.50 (0.63)	7.75 (1.20)
Leiter LA 2014	NCT01042977	24 weeks	Dapagliflozin 10mg	480	63.9 (7.6)	161 (66.9)	94.5(18.7)	8.0 (0.8)	9.2 (2.5)
			Placebo	482	63.6 (7.0)	163 (67)	93.2 (16.8)	8.1 (0.8)	9.0 (2.58)
Yang W 2016	NCT01095666	24 weeks	Dapagliflozin 5mg	147	53.1(9.2)	67 (45.6)	70.8(12.2)	8.09 (0.72)	9.0 (2.2)
			Dapagliflozin 10mg	152	54.6(9.5)	88 (57.9)	71.4 (12.0)	8.17 (0.84)	9.0 (2.2)
			Placebo	145	53.5(9.2)	86 (59.3)	70.9(11.4)	8.13 (0.85)	9.2 (2.5)
Weber MA 2016	NCT01137474	12 weeks	Dapagliflozin 10mg	302	56.2(8.9)	123 (40.7)	86.0(18.4)	8.1(1.0)	8.78(2.43)
			Placebo	311	55.6(8.4)	140 (45.0)	84.1(17.5)	8.0(0.9)	8.92(2.41)
Schumm-Draeger PM 2015	NCT01217892	16 weeks	Dapagliflozin 10mg	99	58.5 (9.8)	49 (49.5)	NA	7.71 (0.71)	8.62 (2.01)
			Placebo	101	58.5 (9.4)	47 (46.5)	NA	7.94 (0.85)	8.76 (1.99)
Matthaei S 2015	NCT01392677	24 weeks	Dapagliflozin 10mg	108	61.1 (9.7)	62 (57.4)	88.6 (17.6)	8.08 (0.91)	9.29 (2.40)
			Placebo	108	60.9 (9.2)	48 (44.4)	90.1 (16.2)	8.24 (0.87)	10.00 (2.39)
Eriksson JW 2018	NCT02279407	12 weeks	Dapagliflozin 10mg	21	65.0 (6.5)	7(33.3)	90.2 (8.7)	7.38 (0.56)	8.99 (1.85)
			Placebo	21	65.6 (6.1)	4(19)	93.0 (12.2)	7.44 (0.80)	9.40 (1.65)
Fadini GP 2017	NCT02327039	12 weeks	Dapagliflozin 10mg	15	61.0(1.8)	10(68.8)	NA	8.2 (0.2)	NA
			Placebo	16	66.3 (1.8)	11(66.7)	NA	8.2 (0.2)	NA
Fioretto P 2019	NCT02413398	24 weeks	Dapagliflozin 10mg	160	65.3 (66.0)	91 (56.5)	88.3 (16.2)	8.03 (1.08)	9.6 (3.0)
			Placebo	161	66.2 (68.0)	91 (56.9)	92.4 (16.8)	8.33 (1.08)	10.1 (3.7)
Yang W 2018	NCT02096705	24 weeks	Dapagliflozin 10mg	139	56.5 (8.39)	66 (47.5)	71.1 (12.0)	8.52 (0.76)	9.96(2.56)
			Placebo	133	58.6 (8.91)	64 (48.1)	72.4 (13.1)	8.58 (0.81)	9.312.24)
Inagaki N 2016	NCT02220920	16 weeks	Canagliflozin 100 mg	76	59.7 (9.4)	44 (57.9)	69.95 (13.93)	8.89 (0.81)	9.44(2.47)
			Placebo	70	56.1 (10.9)	49 (70.0)	69.68 (13.13)	8.85 (0.84)	9.40(2.92)
Inagaki N 2014	NCT01413204	24 weeks	Canagliflozin 100 mg	90	58.4 (10.4)	59 (65.6)	69.10 (14.48)	7.98(0.73)	8.76(1.98)
			Placebo	93	58.2 (11.0)	60 (64.5)	68.57 (15.15)	8.04(0.70)	9.05(1.81)
Lavalle-González FJ 2013	NCT01106677	26 weeks	Canagliflozin 100 mg	368	55.5(9.4)	174 (47.3)	88.8(22.2)	8.0(0.9)	9.1(2.1)
			Canagliflozin 300 mg	367	55.3(9.2)	165 (45.0)	85.4(20.9)	7.9(0.9)	9.3(2.3)
			Placebo	183	55.3(9.8)	94 (51.4)	86.6(22.4)	7.9(0.9)	9.6(2.5)
Wilding JP 2013	NCT01106625	26 weeks	Canagliflozin 100 mg	157	57.4 (10.5)	76 (48.4)	93.8 (22.6)	8.1 (0.9)	9.6 (2.3)
			Canagliflozin 300 mg	157	56.1 (8.9)	87 (55.8)	93.5 (22.0)	8.1 (0.9)	9.3 (2.1)
			Placebo	156	56.8 (8.3)	76 (48.7)	91.2 22.6	8.1 (0.9)	9.4 (2.2)
Rosenstock J 2012	NCT00642278	12 weeks	Canagliflozin 100 mg	64	51.7 (8.0)	36 (56)	87.7 (15.5)	7.83 (0.96)	9.33(2.33)
			Canagliflozin 300 mg	64	52.3(6.9)	36 (56)	87.3(15.9)	7.69 (1.02)	8.83(2.44)
			Placebo	65	53.3(7.8)	31 (48)	85.9(19.5)	7.75 (0.83)	9.11(2.11)
Forst T 2014	NCT01106690	26 weeks	Canagliflozin 100 mg	113	56.7 (10.4)	77 (68.1)	94.2 (22.2)	8.0 (0.9)	9.4 (2.2)
			Canagliflozin 300 mg	114	57.0 (10.2)	63 (55.3)	94.4 (25.9)	7.9 (0.9)	9.1 (2.3)
			Placebo	115	58.3 (9.6)	76 (66.1)	93.8 (22.4)	8.0 (1.0)	9.1 (2.2)
Ji L 2015	NCT01381900	18 weeks	Canagliflozin 100 mg	223	56.5 (8.3)	124 (55.6)	69.1 (11.8)	8.0 (0.9)	8.7 (1.9)
			Canagliflozin 300 mg	227	56.4 (9.2)	113 (49.8)	69.6 (11.9)	8.0 (0.9)	8.9 (2.0)
			Placebo	226	55.8 (9.4)	125 (55.3)	68.6 (11.9)	7.9 (0.9)	8.8 (1.8)
Stenlöf K 2013	NCT01081834	26 weeks	Canagliflozin 100 mg	195	55.1 (10.8)	114 (58.5)	85.8 (21.4)	8.1 (1.0)	9.6 (2.4)
			Canagliflozin 300 mg	197	55.3 (10.2)	108 (54.8)	86.9 (20.5)	8.0 (1.0)	9.6 (2.4)
			Placebo	192	55.7 (10.9)	104 (54.2)	87.6 (19.5)	8.0 (1.0)	9.3 (2.1)
Yale JF 2014	NCT01064414	26 weeks	Canagliflozin 100 mg	90	69.5 (8.2)	58 (64.4)	90.5 (18.4)	7.9 (0.9)	9.4 (2.6)
			Canagliflozin 300 mg	89	67.9 (8.2)	48 (53.9)	90.2 (18.1)	8.0 (0.8)	8.8 (3.2)
			Placebo	90	68.2 (8.4)	57 (63.3)	92.8 (17.4)	8.0 (0.9)	8.9 (2.4)
Inagaki N 2013	NCT01022112	12 weeks	Canagliflozin 100 mg	74	57.7 (10.5)	52 (70.3)	68.61 (14.86)	8.05(0.86)	8.94(1.77)
			Canagliflozin 300 mg	75	57.1 (10.1)	55 (73.3)	71.30 (12.19)	8.17(0.81)	9.40(1.9)
			Placebo	75	57.7 (11.0)	54 (72.0)	72.56(15.36)	7.99(0.77)	9.48(1.77)
Sone H 2020	NCT02589639	16 weeks	Empagliflozin 10mg	86	58.3 (10.0	63 (73.3)	73.3 (11.5)	8.8 (0.7)	9.38(2.40)
			Empagliflozin 25mg	90	58.6 (9.5	61 (67.8)	72.2 (11.4)	8.7 (0.7)	8.67(2.09)
			Placebo	90	59.1 (10.7	69 (76.7)	74.0 (11.3)	8.7 (0.7)	8.84(2.14)
Roden M 2015	NCT01289990	24 weeks	Empagliflozin 10mg	224	56.2 (11.6)	142 (63.4)	78.4 (18.7)	7.87(0.88)	8.5 (1.8)
			Empagliflozin 25mg	224	53.8 (11.6)	145 (64.7)	77.8 (18.0)	7.86(0.85)	8.5 (1.9)
			Placebo	228	54.9 (10.9)	123 (53.9)	78.2 (19.9)	7.91(0.78)	8.6 (2.0)
Kadowaki T 2014	NCT01193218	12 weeks	Empagliflozin 10mg	109	57.9(9.4)	77(70.6)	68.1(14.6)	8.71(1.4)	7.93(0.4)
			Empagliflozin 25mg	109	57.2(9.7)	84(77.1)	68.3(14.1)	8.67(1.87)	7.93(0.65)
			Placebo	109	58.7(8.7)	80(73.4)	69.0(12.2)	8.68(0.22)	7.94(0.3)
Roden M 2013	NCT01177813	12 weeks	Empagliflozin 10mg	224	56.2 (11.6)	142 (63)	78.4 (18.7)	7.87(0.88)	8.48 (1.79)
			Empagliflozin 25mg	224	53.8 (11.6)	145 (65)	77.8 (18.0)	7.86 (0.85)	8.47 (1.89)
			Placebo	228	54.9 (10.9)	123 (54)	78.2 (19.9)	7.91 (0.78)	8.59 (1.99)
Häring HU 2013	NCT01159600	24 weeks	Empagliflozin 10mg	225	57.0 (9.2)	113 (50)	77.1 (18.3)	8.07 (0.81)	8.38 (1.82)
			Empagliflozin 25mg	216	57.4 (9.3)	114 (53)	77.5 (18.8)	8.10 (0.83)	8.69 (1.87)
			Placebo	225	56.9 (9.2)	112 (50)	76.2 (16.9)	8.15 (0.83)	8.42 (1.99)
Ferrannini E 2013	NCT00789035	12 weeks	Empagliflozin 10mg	81	58.0 (30–76)	40 (49.4)	76.8 (45.5–118.0)	8.0 (0.8)	9.9 (2.6)
			Empagliflozin 25mg	82	57.0 (30–79)	41 (50.0)	81.2 (49.1–130.0)	7.8 (0.8)	9.5 (1.4)
			Placebo	82	58.0 (28–80)	45 (54.9)	82.2 (49.0–152.3)	7.8 (0.8)	9.5 (2.2)
Rosenstock J 2013	NCT00749190	12 weeks	Empagliflozin 10mg	71	59 (9.0)	33 (47)	87.9 (14.4)	7.9 (0.7)	9.61(2)
			Empagliflozin 25mg	70	59 (8.1)	37 (53)	90.5 (16.9)	8.1 (0.8)	9.0(2.67)
			Placebo	71	60 (8.5)	33 (47)	87.7 (15.7)	8.0 (0.7)	9.67(2.22)
Ross S 2015	eudract number 2012-000905-53	16 weeks	Empagliflozin 10mg	214	NA	NA	89.10(1.30)	7.83(0.05)	8.9(0.2)
			Empagliflozin 25mg	214	NA	NA	88.72(1.27)	7.73(0.05)	8.7(0.1)
			Placebo	107	NA	NA	90.10(1.78)	7.69(0.07)	8.9(0.2)
Ji L 2019	NCT02630706	26 weeks	Ertugliflozin 5 mg	170	56.1(9.0)	95 (55.9)	71.4 (11.1)	8.1 (0.9)	9.29(2.28)
			Ertugliflozin 15 mg	169	56.3(9.3)	98 (58.0)	69.5 (10.9)	8.1 (0.9)	9.45(2)
			Placebo	167	56.9(9.0)	88 (52.7)	70.1 (12.4)	8.1 (1.0)	9.21(2.09)
Dagogo-Jack S 2018	NCT02036515	26 weeks	Ertugliflozin 5 mg	155	59.2 (9.3)	81 (51.9)	87.6 (18.6)	8.1 (0.9)	9.3 (2.1)
			Ertugliflozin 15 mg	152	59.7 (8.6)	82 (53.6)	86.6 (19.5)	8.0 (0.8)	9.5 (2.2)
			Placebo	152	58.3 (9.2)	100 (65.4)	86.4 (20.8)	8.0 (0.9)	9.4 (2.1)
Rosenstock J 2018	NCT02033889	26 weeks	Ertugliflozin 5 mg	207	56.6 (8.1)	97 (46.9)	84.8 (17.2)	8.1 (0.9)	9.3 (2.5)
			Ertugliflozin 15 mg	205	56.9 (9.4)	93 (45.4)	85.3 (16.5)	8.1 (0.9)	9.3 (2.5)
			Placebo	209	56.5 (8.7)	98 (46.9)	84.5 (17.1)	8.2 (0.9)	9.4 (2.3)
Terra SG 2017	NCT01958671	26 weeks	Ertugliflozin 5 mg	156	56.8 (11.4)	89 (57.1)	94.0 (25.4)	8.16 (0.88)	10.0 (2.7)
			Ertugliflozin 15 mg	152	56.2 (10.8)	90 (59.2)	90.6 (18.3)	8.35 (1.12)	9.9 (2.7)
			Placebo	153	56.1 (10.9)	82 (53.6)	94.2 (25.2)	8.11 (0.92)	10.0 (2.5)
Amin NB 2015	NCT01059825	12 weeks	Ertugliflozin 5 mg	55	54.7(7.7)	31(74.5)	NA	7.88(0.13)	8.69(0.32)
			Placebo	54	54(8.1)	30(55.6)	NA	8.08(0.14)	9.18(0.31)
Grunberger G 2018	NCT01986855	26 weeks	Ertugliflozin 5 mg	158	66.7 (8.3)	84 (53.2)	89.4 (22.5)	8.2 (1.0)	8.94(3.13)
			Ertugliflozin 15 mg	155	67.5 (8.5)	75 (48.4)	85.8 (17.4)	8.2 (0.9)	8.75(2.66)
			Placebo	154	67.5 (8.9)	72 (46.8)	90.4 (18.9	8.1 (0.9)	8.72(3.13)
Cannon CP 2020	NCT01986881	18 weeks	Ertugliflozin 5 mg	2746	64 4 ± 8 1	3862(70.3)	NA	8. 2(1.0)	NA
			Ertugliflozin 15 mg	2747					
			Placebo	2745	64 4 ± 8 0	3806(69.3)	NA	8. 2(0.9)	NA

HbA1c: glycosylated haemoglobin; FPG: fasting plasma glucose.

### 3.2 Quality assessment of included studies

**Figure 2:**
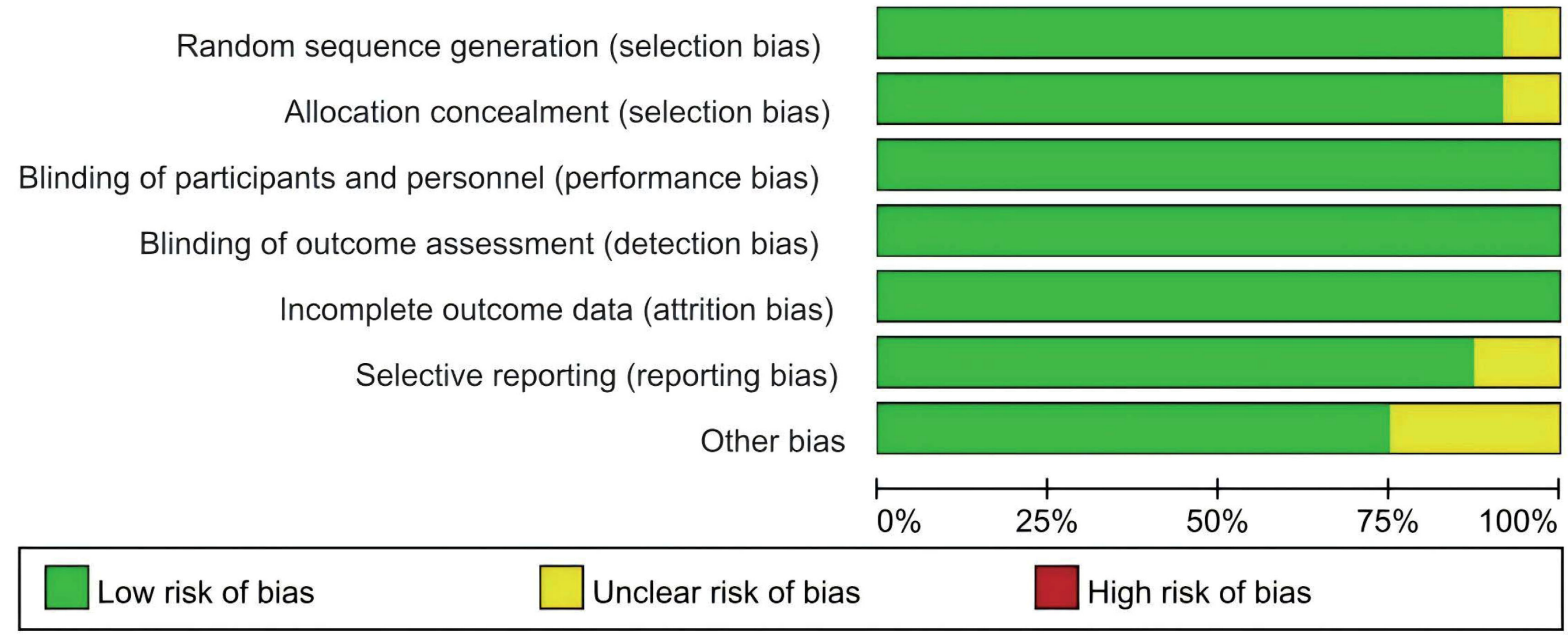
Risk of bias of the included studies.

The risk-of-bias assessment results for the 48 meta-analysis-included studies are presented in **Figure 2**. Four studies presented with an unclear risk of selection bias, whereas low risk was consistently observed across all studies for performance, detection, and attrition bias. Six studies were judged to have an unclear risk of reporting bias, while twelve additional studies exhibited an unclear risk of other forms of bias. Comprehensive details concerning the risk of bias are presented in **Supplementary Table 3**.

### 3.3 Inconsistency analysis

Inconsistency is defined as discrepancies arising between direct and indirect evidence, which may threaten the validity of results derived from network meta-analysis. The node-splitting technique was applied to quantify the level of inconsistency existing between direct and indirect evidence comparisons. Results indicated the absence of both global and local inconsistency, as illustrated in **Supplementary Table 4**.

### 3.4 NMA

#### 3.4.1 HbA1c

**Figure 3:**
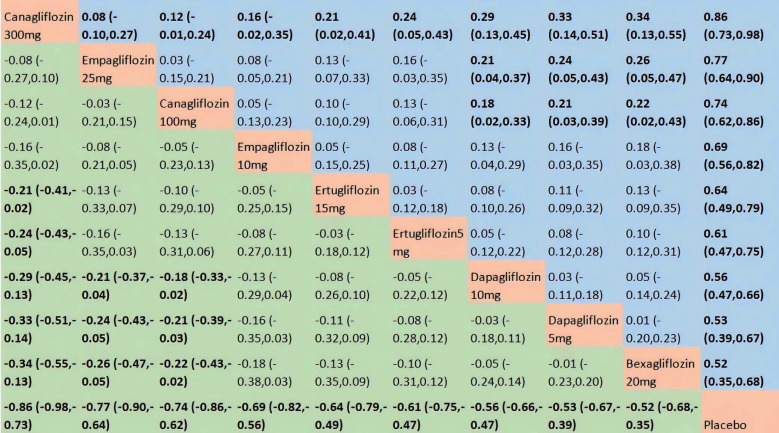
The results of network meta-analysis for HbA1c. The central block of interventions divides the graph into upper and lower triangular sections. In the lower triangle, efficacy estimates represent the ratio of column-defined to row-defined treatments: a MD > 0 favors the column treatment, while a MD < 0 favors the row treatment. The upper triangle is the symmetrical mirror of the lower one. Statistically significant results are in bold.

We analyzed 48 RCTs involving 10 treatment nodes to evaluate their effects on HbA1c (**Supplementary Figure 1A**). Heterogeneity was high, with an I^2^ statistic of 73% and a τ^2^ value of 0.02. Compared with placebo, bexagliflozin significantly improved HbA1c reduction, with a MD of 0.52 (95% CI: 0.35–0.68). Among the active comparators, empagliflozin 25 mg and canagliflozin (300 mg, 100 mg) all induced more substantial HbA1c reductions than bexagliflozin. No other active treatments differed significantly from bexagliflozin in terms of HbA1c-lowering efficacy (**Figure 3**). Bexagliflozin attained a surface under the cumulative ranking curve (SUCRA) value of 0.24 for HbA1c reduction, corresponding to a 9th-place ranking among the 10 treatment nodes. In contrast, canagliflozin 300 mg was the top-ranked intervention with a SUCRA score of 0.96 (**Supplementary Table 5**).

#### 3.4.2 FPG

**Figure 4:**
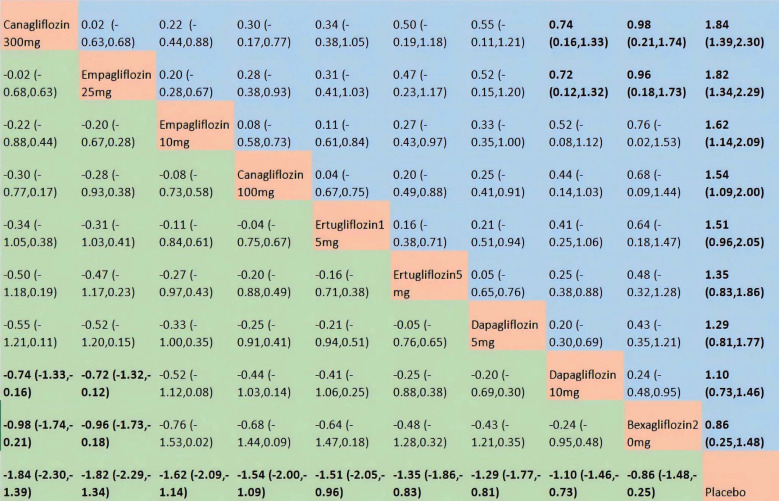
The results of network meta-analysis for FPG. The central block of interventions divides the graph into upper and lower triangular sections. In the lower triangle, efficacy estimates represent the ratio of column-defined to row-defined treatments: a MD > 0 favors the column treatment, while a MD < 0 favors the row treatment. The upper triangle is the symmetrical mirror of the lower one. Statistically significant results are in bold.

A total of 46 RCTs encompassing 10 treatment nodes were analyzed to assess their impacts on the changes of FPG (**Supplementary Figure 1B**). Heterogeneity was high, with an I^2^ statistic of 90% and a τ^2^ value of 0.47. Compared with placebo, bexagliflozin was found to elicit a statistically significant improvement in FPG reduction (MD = 0.86; 95% CI,0.25-1.48). Of the active comparators evaluated, canagliflozin 300 mg and empagliflozin 25 mg produced a slightly greater FPG reduction compared with bexagliflozin. No significant discrepancies in FPG-lowering effectiveness were observed between bexagliflozin and the remaining active interventions (**Figure 4**). For the outcome of FPG reduction, bexagliflozin achieved a SUCRA value of 0.18, which corresponded to a 9th-place ranking across the 10 treatment nodes. Furthermore, canagliflozin 300 mg ranked 1st, achieving a SUCRA value of 0.86 (**Supplementary Table 5**).

#### 3.4.3 Body weight

A total of 44 RCTs involving 10 treatment nodes were analyzed to evaluate their effects on body weight change (**Supplementary Figure 1C).** Heterogeneity was low, with an I^2^ statistic of 39.9% and a τ^2^ value of 0.07. Compared with placebo, bexagliflozin exerted a statistically significant effect on body weight reduction (MD = 2.13; 95% CI,1.70-2.57). Among the active comparators evaluated, bexagliflozin elicited a significantly greater reduction in body weight than dapagliflozin 5 mg, yet a smaller body weight loss effect was observed with bexagliflozin relative to canagliflozin 300 mg. No notable differences in body weight-lowering efficacy were detected between bexagliflozin and the other active interventions (**Supplementary Figure 2**). For body weight reduction outcomes, bexagliflozin attained a SUCRA value of 0.80, placing it 2nd among the 10 treatment nodes. Meanwhile, canagliflozin 300 mg ranked 1st, achieving a SUCRA value of 0.99 (**Supplementary Table 5**).

#### 3.4.4 SBP

A total of 33 RCTs encompassing 10 treatment nodes were analyzed to assess their impacts on SBP (**Supplementary Figure 1D**). Heterogeneity was low, with an I^2^ statistic of 45.6% and a τ^2^ value of 1.31. Compared with placebo, bexagliflozin demonstrated a statistically significant SBP-lowering effect (MD = 3.15; 95% CI,1.27-5.02). No significant discrepancies in SBP reduction efficacy were observed between bexagliflozin and any of the other active interventions (**Supplementary Figure 3**). In terms of SBP reduction outcomes, bexagliflozin achieved a SUCRA value of 0.50 corresponding to a 6th-place ranking among the 10 treatment nodes. Moreover, canagliflozin 300 mg ranked 1st, achieving a SUCRA value of 0.68 (**Supplementary Table 5**).

#### 3.4.5 DBP

A total of 20 RCTs involving 10 treatment nodes were analyzed to assess their effects on DBP (**Supplementary Figure 1E**). Heterogeneity was low, with an I^2^ statistic of 22.6% and a τ^2^ value of 0.15. Compared with placebo, bexagliflozin exhibited a statistically significant DBP-lowering effect (MD = 2.24; 95% CI,0.76-3.73). Among the assessed active comparators, bexagliflozin produced a markedly more pronounced reduction in DBP than empagliflozin 10 mg. No significant discrepancies in DBP reduction efficacy were identified between bexagliflozin and the remaining active interventions (**Supplementary Figure 4**). For the DBP reduction outcome, bexagliflozin achieved a SUCRA value of 0.72, which placed it third among the 10 treatment nodes. Notably, ertugliflozin 15 mg ranked 1st, achieving a SUCRA value of 0.69 (**Supplementary Table 5**).

#### 3.4.6 Safety analysis

For hypoglycemia incidence, 30 RCTs covering 10 treatment nodes were analyzed (**Supplementary Figure 1F**), with low heterogeneity (I^2^=44.9%, τ^2^=0.15); no statistically significant differences were observed between bexagliflozin and active comparators or placebo (**Supplementary Figure 5**). For urinary tract infection, 41 RCTs involving 10 treatment nodes were included (**Supplementary Figure 1G**), with no heterogeneity detected (τ^2^=0, I^2^=0); dapagliflozin (5 mg, 10 mg) had a higher incidence than bexagliflozin, and no significant differences were noted between bexagliflozin and other comparators (active agents and placebo) (**Supplementary Figure 6**). For genital mycotic infection, 35 RCTs with 10 treatment nodes were analyzed (**Supplementary Figure 1H**), showing low heterogeneity (I^2^=4.9%, τ^2^=0.02); bexagliflozin did not show statistically significant differences from the above comparators, while ertugliflozin (5 mg, 15 mg), canagliflozin (100 mg, 300 mg) and dapagliflozin 5 mg were associated with an elevated incidence compared to placebo, with no significant differences in all other pairwise comparisons (**Supplementary Figure 7**).

### 3.5 Certainty of evidence

**Supplementary Table 6** summarizes the level of confidence in the evidence for each of the seven outcomes. For all outcomes, low or very low confidence was assigned to the evidence for 36.1% of placebo comparisons (**Supplementary Figure 8**); this pattern was even more pronounced for pairwise drug comparisons, where 87.5% were rated as such (**Supplementary Table 6**). These low certainty ratings were predominantly attributed to imprecision, heterogeneity, and incoherence across the analyses.

### 3.6 Publication bias

Funnel plot and Egger’s regression test findings are reported in **Supplementary Table 7** and **Supplementary Figure 9**. For most efficacy and safety outcomes, funnel plots corresponding to body weight, DBP, hypoglycemia, urinary tract infection and genital mycotic infection displayed approximate symmetry, with all associated p-values above 0.05. Conversely, notable evidence of publication bias was detected for HbA1c, FPG and SBP outcomes.

### 3.7 Sensitivity analysis

For HbA1c reduction, sensitivity analysis excluding heterogeneity-driven studies (18^36^ and 45^63^) included a total of 46 studies; the corresponding SUCRA results are presented in **Supplementary Figure 10**. This analysis yielded outcomes consistent with the primary findings for the majority of interventions. Separate sensitivity analysis for HbA1c reduction, which excluded small-sample studies (10^28^, 12^30^ and 18^36^), also incorporated 46 studies, with the associated SUCRA results shown in **Supplementary Figure 11**. Findings from this second analysis were likewise consistent with the main analysis across most interventions.

## 4 Discussion

This NMA delivers an updated synthesis of the current evidence base to quantify the relative efficacy and safety of bexagliflozin versus other SGLT2 inhibitors in patients with T2DM. Conducted on the basis of 48 RCTs, this analysis included five SGLT2 inhibitors, ten interventions, and a total of 26,838 participants. Overall, the quality of evidence was predominantly rated as low risk of bias, with a low proportion of studies presenting unclear risk of bias. Our findings demonstrated that bexagliflozin yielded statistically significant improvements in HbA1c reduction, FPG reduction, and body weight loss relative to placebo; additionally, this agent was associated with a significant reduction in both SBP and DBP. In terms of HbA1c reduction, canagliflozin 300 mg, empagliflozin 25 mg and canagliflozin 100 mg all achieved more substantial reductions than bexagliflozin; no significant differences were noted in this outcome between bexagliflozin and all other SGLT2 inhibitors, with comparable HbA1c-lowering effects observed. For FPG reduction, canagliflozin 300 mg and empagliflozin 25 mg elicited a slightly greater decrease relative to bexagliflozin, whereas bexagliflozin exhibited similar FPG-lowering efficacy to that of the remaining SGLT2 inhibitors with no statistically significant discrepancies detected. Regarding body weight reduction, bexagliflozin was associated with a significantly greater decrease than dapagliflozin 5 mg, while showing comparable weight-lowering efficacy relative to all other active SGLT2 inhibitors. Although bexagliflozin ranked second for body weight reduction based on SUCRA values, interpretation of effect sizes and 95% confidence intervals revealed that its benefit was statistically significant only in comparison with dapagliflozin 5 mg. Taken together, these findings suggest that bexagliflozin may confer a relatively favorable benefit with respect to body weight reduction. Moreover, bexagliflozin induced a significantly greater reduction in DBP than empagliflozin 10 mg, while its SBP and DBP-lowering efficacy was comparable to that of all other active SGLT2 inhibitor interventions, with no statistically significant differences observed in either SBP or DBP reduction between bexagliflozin and these comparators. For safety analysis, dapagliflozin (5 mg, 10 mg) carried an increased incidence of urinary tract infection than bexagliflozin, with no other significant differences observed in this outcome between bexagliflozin and both active agents and placebo. Furthermore, we strengthened the certainty of evidence by applying the CINeMA methodology for evidence quality assessment, supplemented by tabular result display.

Our findings align with the conclusions of prior systematic reviews and meta-analyses, demonstrating that dapagliflozin, empagliflozin, canagliflozin and ertugliflozin yielded significant reductions in HbA1c, FPG and overall body weight relative to placebo. Additionally, the safety profile of dapagliflozin, empagliflozin, canagliflozin and ertugliflozin was consistent with that observed in the placebo group (68–71). Our study, however, extends beyond the current body of literature through a more comprehensive analysis of the therapeutic effects of these agents, while also incorporating bexagliflozin—a novel agent approved by the FDA for T2DM in January 2023. We adopted a NMA approach to quantitatively compare the efficacy of distinct intervention dosages. This analytical approach facilitated the robust characterization of interventional disparities in efficacy and safety across a broad range of clinical outcomes. First, our findings demonstrated that bexagliflozin was more effective than placebo at reducing HbA1c, FPG, SBP, DBP and overall body weight, which is consistent with the conclusions of prior research. These prior studies have reported that bexagliflozin yields a marked reduction in HbA1c levels among patients with T2DM relative to the placebo group, with no statistically significant adverse events observed (10, 72). As far as we are aware, no prior research has investigated the efficacy and safety of bexagliflozin compared with active comparators. Notably, this NMA is the first study to explore the differences between bexagliflozin and other SGLT2 inhibitors, thereby providing additional evidence to clarify the efficacy and safety profile of bexagliflozin. Regarding efficacy, bexagliflozin had comparable efficacy in HbA1c and FPG reduction, with no statistically significant differences against ertugliflozin (5 mg, 15 mg), dapagliflozin (5 mg, 10 mg), and empagliflozin 10 mg for both endpoints. For body weight loss, bexagliflozin induced a significantly greater reduction than dapagliflozin 5 mg but a markedly weaker effect than canagliflozin 300 mg; no notable discrepancies were detected with the remaining active interventions canagliflozin 100 mg, ertugliflozin (5 mg, 15 mg), empagliflozin (10 mg, 25 mg) and dapagliflozin 10 mg. Bexagliflozin showed comparable SBP-lowering effects to all evaluated dosages of dapagliflozin, empagliflozin, canagliflozin and ertugliflozin, and equivalent DBP reduction efficacy versus ertugliflozin (5 mg, 15 mg), canagliflozin (100 mg, 300 mg), empagliflozin 25 mg and dapagliflozin (5 mg, 10 mg), with no statistically significant differences noted in pairwise comparisons for DBP. Based on SUCRA values and pairwise comparison results, canagliflozin 300 mg exhibited the optimal efficacy for HbA1c reduction, FPG reduction, and body weight loss. Shyangdan DS et al. (73) reported that canagliflozin 300 mg exhibited the greatest efficacy in HbA1c and body weight reduction among four interventions (canagliflozin 100 mg, 300 mg; empagliflozin 10 mg, 25 mg). Zaccardi et al. (74) concluded that canagliflozin 300 mg reduced HbA1c and FPG to a greater extent than dapagliflozin and empagliflozin. These findings align with our results, which confirm that canagliflozin 300 mg demonstrates the greatest efficacy among SGLT2 inhibitors. Notably, our study additionally included the novel SGLT2 inhibitor bexagliflozin, and still identified canagliflozin 300 mg as the most effective intervention in glycemic and weight control. In terms of safety, dapagliflozin, empagliflozin, bexagliflozin, canagliflozin, and ertugliflozin exhibited no differences from placebo in the risk of hypoglycemia. This phenomenon can be attributed to the glucosuric effect of SGLT2 inhibitors being dependent on the filtered glucose load: these agents lose their glucose-lowering efficacy when the filtered glucose load falls below 80 g per day (75). In our analysis, all included SGLT2 inhibitors except dapagliflozin exhibited no significant differences from placebo in the risk of urinary tract infection; this finding is consistent with the conclusions of a prior study (76), which reported that SGLT2 inhibitors do not elevate the risk of urinary tract infection relative to placebo, while higher doses of dapagliflozin carried an elevated incidence of this adverse event. Moreover, we found that only dapagliflozin 5 mg, ertugliflozin (5 mg, 15 mg), and canagliflozin (100 mg, 300 mg) were associated with an elevated incidence of genital mycotic infection. This finding is consistent with the conclusions of two safety meta-analyses (76, 77), which also reported that SGLT-2 inhibitors confer an increased risk of genital tract infections, with canagliflozin and dapagliflozin posing the highest risk in particular.

This NMA has a solid evidence base and strict methodology. However, it still has some limitations. Sensitivity analyses excluding studies that exerted the most significant influence on heterogeneity yielded generally consistent findings with the primary analysis, supporting the robustness and reliability of the main conclusions. First, substantial heterogeneity was detected across several efficacy outcomes, most prominently for HbA1c and FPG. Treatment duration and concomitant background antidiabetic therapy were considered major potential sources of heterogeneity. Specifically, the included studies featured variable treatment durations of 12, 16, and 24 weeks, while background antidiabetic regimens also differed, including metformin monotherapy, drug-naive populations, and other antidiabetic treatments. To address this heterogeneity, we conducted a sensitivity analysis by excluding studies that exerted the most pronounced influence on heterogeneity. Sensitivity analyses following the exclusion of these highly influential studies yielded results generally consistent with those of the primary analysis, thereby supporting the robustness and reliability of the main findings. According to CINeMA, most pairwise comparisons showed low to very low confidence. Funnel plots and Egger’s test also suggested potential publication bias. For HbA1c, FPG, and SBP, this bias may come from the over-reporting of positive results in published studies. Unpublished data were less included. This imbalance can make the pooled effect sizes larger than they really are. It may overestimate the efficacy of each drug and widen the differences between treatments. Therefore, the results should be interpreted carefully. Second, some included studies had small sample sizes (<100 patients). Small trials are prone to produce unstable and inflated effect estimates. We performed a sensitivity analysis by removing these small studies. The main results did not change obviously. This supports the reliability of our conclusions. Third, all data were obtained from published literature only. Our findings may be updated when more high-quality evidence becomes available. Fourth, other safety endpoints associated with SGLT2 inhibitors, such as ketoacidosis, volume depletion, acute kidney injury, fractures, amputations, were not assessed in this study. This represents a limitation, as insufficient reporting of these outcomes across the included trials prevented reliable comparative analysis. In addition, more large, well-designed RCTs with active controls are needed to confirm our results.

Bexagliflozin exhibited efficacy comparable to that of most SGLT2 inhibitors in reducing HbA1c, lowering FPG, decreasing body weight, and reducing SBP and DBP—including ertugliflozin (5 mg, 15 mg), empagliflozin 10 mg, dapagliflozin (5 mg, 10 mg)—and yielded a greater reduction in body weight relative to dapagliflozin 5 mg. In terms of clinical safety, bexagliflozin had a favorable profile compared with other SGLT2 inhibitors and placebo. Furthermore, canagliflozin 300 mg may serve as a strong effective therapeutic option in glycemic and weight control for patients with T2DM among available SGLT2 inhibitors. Collectively, the present analyses confirm that bexagliflozin is an efficacious therapeutic choice for the management of T2DM, with a safety profile consistent with that of other SGLT2 inhibitors.

## Data Availability

The original contributions presented in the study are included in the article/**Supplementary Material**. Further inquiries can be directed to the corresponding author.
