# Supplementary material for: Comparison of effectiveness and safety of bexagliflozin and other sodium-glucose cotransporter 2 inhibitors for type 2 diabetes mellitus in adults: systematic review and network meta-analysis of randomized controlled trials
Source: Front Endocrinol (Lausanne). 2026 May 13;17:1843370. doi: 10.3389/fendo.2026.1843370 (PMC13212240; doi:10.3389/fendo.2026.1843370)

Supplementary Material

**Table S1.** PRISMA NMA Checklist

| **Section and Topic** | **Item #** | **Checklist item** | **Location where item is reported** |
| --- | --- | --- | --- |
| **TITLE** | | |  |
| Title | 1 | Identify the report as a systematic review. | 1 |
| **ABSTRACT** | | |  |
| Abstract | 2 | See the PRISMA 2020 for Abstracts checklist. | 1 |
| **INTRODUCTION** | | |  |
| Rationale | 3 | Describe the rationale for the review in the context of existing knowledge. | 1 |
| Objectives | 4 | Provide an explicit statement of the objective(s) or question(s) the review addresses. | 1 |
| **METHODS** | | |  |
| Eligibility criteria | 5 | Specify the inclusion and exclusion criteria for the review and how studies were grouped for the syntheses. | 3 |
| Information sources | 6 | Specify all databases, registers, websites, organisations, reference lists and other sources searched or consulted to identify studies. Specify the date when each source was last searched or consulted. | 3 |
| Search strategy | 7 | Present the full search strategies for all databases, registers and websites, including any filters and limits used. | Table S2 |
| Selection process | 8 | Specify the methods used to decide whether a study met the inclusion criteria of the review, including how many reviewers screened each record and each report retrieved, whether they worked independently, and if applicable, details of automation tools used in the process. | 3 |
| Data collection process | 9 | Specify the methods used to collect data from reports, including how many reviewers collected data from each report, whether they worked independently, any processes for obtaining or confirming data from study investigators, and if applicable, details of automation tools used in the process. | 3 |
| Data items | 10a | List and define all outcomes for which data were sought. Specify whether all results that were compatible with each outcome domain in each study were sought (e.g. for all measures, time points, analyses), and if not, the methods used to decide which results to collect. | 3 |
|  | 10b | List and define all other variables for which data were sought (e.g. participant and intervention characteristics, funding sources). Describe any assumptions made about any missing or unclear information. | 3 |
| Study risk of bias assessment | 11 | Specify the methods used to assess risk of bias in the included studies, including details of the tool(s) used, how many reviewers assessed each study and whether they worked independently, and if applicable, details of automation tools used in the process. | 3-4 |
| Effect measures | 12 | Specify for each outcome the effect measure(s) (e.g. risk ratio, mean difference) used in the synthesis or presentation of results. | 4 |
| Synthesis methods | 13a | Describe the processes used to decide which studies were eligible for each synthesis (e.g. tabulating the study intervention characteristics and comparing against the planned groups for each synthesis (item #5)). | 3 and Fig. 1 |
|  | 13b | Describe any methods required to prepare the data for presentation or synthesis, such as handling of missing summary statistics, or data conversions. | 4 |
|  | 13c | Describe any methods used to tabulate or visually display results of individual studies and syntheses. | 4 |
|  | 13d | Describe any methods used to synthesize results and provide a rationale for the choice(s). If meta-analysis was performed, describe the model(s), method(s) to identify the presence and extent of statistical heterogeneity, and software package(s) used. | 4 |
|  | 13e | Describe any methods used to explore possible causes of heterogeneity among study results (e.g. subgroup analysis, meta-regression). | 4 |
|  | 13f | Describe any sensitivity analyses conducted to assess robustness of the synthesized results. | 5 |
| Reporting bias assessment | 14 | Describe any methods used to assess risk of bias due to missing results in a synthesis (arising from reporting biases). | 4-5 |
| Certainty assessment | 15 | Describe any methods used to assess certainty (or confidence) in the body of evidence for an outcome. | 4 |
| **RESULTS** | | |  |
| Study selection | 16a | Describe the results of the search and selection process, from the number of records identified in the search to the number of studies included in the review, ideally using a flow diagram. | 5 |
|  | 16b | Cite studies that might appear to meet the inclusion criteria, but which were excluded, and explain why they were excluded. | Not cited due to too many studies |
| Study characteristics | 17 | Cite each included study and present its characteristics. | Table 1 |
| Risk of bias in studies | 18 | Present assessments of risk of bias for each included study. | 5, Fig. 2,Table S3 |
| Results of individual studies | 19 | For all outcomes, present, for each study: (a) summary statistics for each group (where appropriate) and (b) an effect estimate and its precision (e.g. confidence/credible interval), ideally using structured tables or plots. | Fig. 3-7, Fig. S2-S4, 5-7 |
| Results of syntheses | 20a | For each synthesis, briefly summarise the characteristics and risk of bias among contributing studies. | Table 1 |
|  | 20b | Present results of all statistical syntheses conducted. If meta-analysis was done, present for each the summary estimate and its precision (e.g. confidence/credible interval) and measures of statistical heterogeneity. If comparing groups, describe the direction of the effect. | Fig. 3-7, Fig. S2-S4, 5-7 |
|  | 20c | Present results of all investigations of possible causes of heterogeneity among study results. | 5-7 |
|  | 20d | Present results of all sensitivity analyses conducted to assess the robustness of the synthesized results. | 7 |
| Reporting biases | 21 | Present assessments of risk of bias due to missing results (arising from reporting biases) for each synthesis assessed. | 7,Fig. S6 |
| Certainty of evidence | 22 | Present assessments of certainty (or confidence) in the body of evidence for each outcome assessed. | Table S5  Fig. S5 |
| **DISCUSSION** | | |  |
| Discussion | 23a | Provide a general interpretation of the results in the context of other evidence. | 7 |
|  | 23b | Discuss any limitations of the evidence included in the review. | 7-8 |
|  | 23c | Discuss any limitations of the review processes used. | 8 |
|  | 23d | Discuss implications of the results for practice, policy, and future research. | 8 |
| **OTHER INFORMATION** | | |  |
| Registration and protocol | 24a | Provide registration information for the review, including register name and registration number, or state that the review was not registered. | CRD420251025292 |
|  | 24b | Indicate where the review protocol can be accessed, or state that a protocol was not prepared. | PROSPERO |
|  | 24c | Describe and explain any amendments to information provided at registration or in the protocol. | No |
| Support | 25 | Describe sources of financial or non-financial support for the review, and the role of the funders or sponsors in the review. | 9 |
| Competing interests | 26 | Declare any competing interests of review authors. | 9 |
| Availability of data, code and other materials | 27 | Report which of the following are publicly available and where they can be found: template data collection forms; data extracted from included studies; data used for all analyses; analytic code; any other materials used in the review. | 9 |

*From:*  Page MJ, McKenzie JE, Bossuyt PM, Boutron I, Hoffmann TC, Mulrow CD, et al. The PRISMA 2020 statement: an updated guideline for reporting systematic reviews. BMJ 2021;372:n71. doi: 10.1136/bmj.n71

| **Table S2.Literature Search Strategy** | |
| --- | --- |
| **Pubmed** | #1 SGLT 2 inhibitor*[Title/Abstract] OR sodium-glucose transporter 2 inhibitors* OR bexagliflozin[Title/Abstract] OR canagliflozin[Title/Abstract] OR dapagliflozin[Title/Abstract] OR empagliflozin[Title/Abstract] OR ertugliflozin[Title/Abstract]  #2 "Diabetes Mellitus, Type 2"[Mesh] OR "diabetes mellitus"[Title/Abstract] OR "type 2 diabetes"[Title/Abstract] OR "type II diabetes"[Title/Abstract] OR "non-insulin dependent diabetes"[Title/Abstract] OR "NIDDM"[Title/Abstract] OR "Diabetes Mellitus, Maturity Onset"[Title/Abstract] OR "type 2 diabetes mellitus"[Title/Abstract] OR "adult-onset diabetes mellitus"[Title/Abstract]  #3 #1 AND #2  #4 #3 AND Filters: Randomized Controlled Trial |
| **Web of Science** | (((TS=( “Diabetes mellitus” or “type 2 diabetes” or “type II diabetes” or “non-insulin dependent diabetes” or “NIDDM” or “Diabetes Mellitus, Maturity Onset” or “Type 2 Diabetes Mellitus” or “Adult-Onset Diabetes Mellitus”)) AND TS=(“sodium glucose co-transporter-2 inhibitor” OR SGLT2 inhibitor OR SGLT2i OR canagliflozin OR dapagliflozin OR empagliflozin OR ertugliflozin OR bexagliflozin)) AND TS= Clinical Trial (Filter)) |
| **ClinicalTrials.gov** | Condition or disease: “Diabetes mellitus” OR “type 2 diabetes” OR “type II diabetes” OR “non-insulin dependent diabetes” OR “NIDDM” OR “Diabetes Mellitus, Maturity Onset” OR “Type 2 Diabetes Mellitus” OR “Adult-Onset Diabetes Mellitus”  Intervention/Treatment：(”SGLT2 inhibitors” OR “sodium-glucose transporter 2 inhibitors” OR canagliflozin OR dapagliflozin OR empagliflozin OR ertugliflozin OR bexagliflozin) |
| **Embase** | #1 'SGLT-2 inhibitor*':ab,ti OR 'bexagliflozin':ab,ti OR 'ertugliflozin':ab,ti OR 'empagliflozin':ab,ti OR 'dapagliflozin':ab,ti OR 'canagliflozin':ab,ti OR 'sodium-glucose transporter 2 inhibitors*':ab,ti  #2 ('diabetes mellitus':ab,ti OR 'type 2 diabetes':ab,ti OR 'type ii diabetes':ab,ti OR 'non-insulin dependent diabetes':ab,ti OR 'niddm':ab,ti OR 'diabetes mellitus, maturity onset':ab,ti OR 'type 2 diabetes mellitus':ab,ti OR 'non insulin dependent diabetes mellitus':ab,ti)  #3 #1 AND #2  #4 #3 AND 'randomized controlled trial'/de |

#

**Table S3. Risk of bias**

| **Study** | **Random sequence generation** | **Allocation concealment** | **Blinding of participants and personnel** | **Blinding of outcome assessment** | **Incomplete outcome data** | **Other source of bias** |
| --- | --- | --- | --- | --- | --- | --- |
| Halvorsen YC 2019 | Low risk | Low risk | Low risk | Low risk | Low risk | Low risk |
| Halvorsen YD 2020 | Low risk | Low risk | Low risk | Low risk | Low risk | Low risk |
| Allegretti AS 2019 | Low risk | Low risk | Low risk | Low risk | Low risk | Low risk |
| Lock JP 2016 | Low risk | Low risk | Low risk | Low risk | Low risk | Low risk |
| Lock JP 2015 | Low risk | Low risk | Low risk | Low risk | Low risk | Low risk |
| Lock JP 2017 | Low risk | Low risk | Low risk | Low risk | Low risk | Low risk |
| Bailey CJ 2012 | Low risk | Low risk | Low risk | Low risk | Low risk | Low risk |
| Kaku K 2013 | Low risk | Low risk | Low risk | Low risk | Low risk | Low risk |
| Ferrannini E 2010 | Unclear risk | Unclear risk | Low risk | Low risk | Low risk | Low risk |
| Lambers HHJ 2013 | Low risk | Low risk | Low risk | Low risk | Low risk | Low risk |
| List JF 2009 | Unclear risk | Unclear risk | Low risk | Low risk | Low risk | Low risk |
| Bailey CJ 2010 | Low risk | Low risk | Low risk | Low risk | Low risk | Low risk |
| Cefalu WT 2015 | Low risk | Low risk | Low risk | Low risk | Unclear risk | Low risk |
| Kaku K 2014 | Low risk | Low risk | Low risk | Low risk | Unclear risk | Low risk |
| Leiter LA 2014 | Low risk | Low risk | Low risk | Low risk | Unclear risk | Low risk |
| Yang W 2016 | Low risk | Low risk | Low risk | Low risk | Low risk | Low risk |
| Weber MA 2016 | Low risk | Low risk | Low risk | Low risk | Unclear risk | Low risk |
| Schumm-Draeger PM 2015 | Low risk | Low risk | Low risk | Low risk | Low risk | Low risk |
| Matthaei S 2015 | Low risk | Low risk | Low risk | Low risk | Low risk | Low risk |
| Eriksson JW 2018 | Low risk | Low risk | Low risk | Low risk | Low risk | Low risk |
| Fadini GP 2017 | Low risk | Low risk | Low risk | Low risk | Low risk | Low risk |
| Fioretto P 2019 | Low risk | Low risk | Low risk | Low risk | Low risk | Low risk |
| Yang W 2018 | Low risk | Low risk | Low risk | Low risk | Low risk | Low risk |
| Inagaki N 2016 | Low risk | Low risk | Low risk | Low risk | Low risk | Low risk |
| Inagaki N 2014 | Low risk | Low risk | Low risk | Low risk | Unclear risk | Unclear risk |
| Lavalle-González FJ 2013 | Low risk | Low risk | Low risk | Low risk | Low risk | Low risk |
| Wilding JP 2013 | Low risk | Low risk | Low risk | Low risk | Low risk | Low risk |
| Rosenstock J 2012 | Low risk | Low risk | Low risk | Low risk | Low risk | Low risk |
| Forst T 2014 | Unclear risk | Unclear risk | Low risk | Low risk | Low risk | Low risk |
| Ji L 2015 | Low risk | Low risk | Low risk | Low risk | Low risk | Low risk |
| Stenlöf K 2013 | Low risk | Low risk | Low risk | Low risk | Low risk | Low risk |
| Yale JF 2014 | Low risk | Low risk | Low risk | Low risk | Low risk | Low risk |
| Inagaki N 2013 | Low risk | Low risk | Low risk | Low risk | Unclear risk | Unclear risk |
| Sone H 2020 | Low risk | Low risk | Low risk | Low risk | Low risk | Low risk |
| Roden M 2015 | Low risk | Low risk | Low risk | Low risk | Low risk | Low risk |
| Kadowaki T 2014 | Low risk | Low risk | Low risk | Low risk | Unclear risk | Unclear risk |
| Roden M 2013 | Low risk | Low risk | Low risk | Low risk | Low risk | Low risk |
| Häring HU 2013 | Low risk | Low risk | Low risk | Low risk | Low risk | Low risk |
| Ferrannini E 2013 | Low risk | Low risk | Low risk | Low risk | Low risk | Low risk |
| Rosenstock J 2013 | Low risk | Low risk | Low risk | Low risk | Low risk | Low risk |
| Ross S 2015 | Unclear risk | Low risk | Low risk | Low risk | Low risk | Unclear risk |
| Ji L 2019 | Low risk | Low risk | Low risk | Low risk | Low risk | Unclear risk |
| Dagogo-Jack S 2018 | Low risk | Low risk | Low risk | Low risk | Low risk | Unclear risk |
| Rosenstock J 2018 | Low risk | Low risk | Low risk | Low risk | Low risk | Unclear risk |
| Terra SG 2017 | Low risk | Low risk | Low risk | Low risk | Low risk | Unclear risk |
| Amin NB 2015 | Low risk | Low risk | Low risk | Low risk | Low risk | Unclear risk |
| Grunberger G 2018 | Low risk | Low risk | Low risk | Low risk | Low risk | Unclear risk |
| Cannon CP 2020 | Low risk | Low risk | Low risk | Low risk | Low risk | Unclear risk |

# Table S4:Node splitting test for inconsistency

| **Outcome** | **Side** | **Direct** | |  | **Indirect** | |  | **Difference** | |  |  |
| --- | --- | --- | --- | --- | --- | --- | --- | --- | --- | --- | --- |
|  |  | Coef. | Std. Err. |  | Coef. | Std. Err. |  | Coef. | Std. Err. |  | ***P* > \|z** |
| HbA1c | A VS. B | - | - |  | - | - |  | - | - |  | - |
|  | A VS. C | -0.557 | 0.758 |  | -0.354 | 0.191 |  | -0.204 | 0.205 |  | 0.320 |
|  | A VS. D | -0.557 | 0.05 |  | -1.238 | 0.491 |  | 0.681 | 0.494 |  | 0.168 |
|  | A VS. E | - | - |  | - | - |  | - | - |  | - |
|  | A VS. F | -0.837 | 0.637 |  | -1.633 | 0.406 |  | 0.796 | 0.411 |  | 0.053 |
|  | A VS. G | - | - |  | - | - |  | - | - |  | - |
|  | A VS. H | - | - |  | - | - |  | - | - |  | - |
|  | A VS. I | - | - |  | - | - |  | - | - |  | - |
|  | A VS. J | -0.638 | 0.078 |  | -0.819 | 0.413 |  | 0.180 | 0.420 |  | 0.668 |
|  | C VS. D | -0.083 | 0.079 |  | 0.168 | 0.161 |  | -0.251 | 0.180 |  | 0.164 |
|  | E VS. F | -0.135 | 0.064 |  | 0.661 | 0.406 |  | -0.796 | 0.411 |  | 0.053 |
|  | G VS. H | - | - |  | - | - |  | - | - |  | - |
|  | I VS. J | -0.038 | 0.078 |  | 0.142 | 0.413 |  | -0.180 | 0.420 |  | 0.668 |
| FPG | A VS. B | - | - |  | - | - |  | - | - |  | - |
|  | A VS. C | -1.197 | 0.261 |  | -1.985 | 0.699 |  | 0.788 | 0.746 |  | 0.291 |
|  | A VS. D | -1.081 | 0.188 |  | -2.246 | 1.540 |  | 1.165 | 1.552 |  | 0.453 |
|  | A VS. E | - | - |  | - | - |  | - | - |  | - |
|  | A VS. F | -1.827 | 0.236 |  | -2.442 | 1.565 |  | 0.615 | 1.583 |  | 0.698 |
|  | A VS. G | - | - |  | - | - |  | - | - |  | - |
|  | A VS. H | - | - |  | - | - |  | - | - |  | - |
|  | A VS. I | - | - |  | - | - |  | - | - |  | - |
|  | A VS. J | -1.500 | 0.285 |  | -1.696 | 1.528 |  | 0.196 | 1.554 |  | 0.900 |
|  | C VS. D | 0.268 | 0.283 |  | -0.109 | 0.577 |  | 0.378 | 0.643 |  | 0.557 |
|  | E VS. F | -0.315 | 0.245 |  | 0.299 | 1.561 |  | 0.615 | 1.583 |  | 0.698 |
|  | G VS. H | - | - |  | - | - |  | - | - |  | - |
|  | I VS. J | -0.168 | 0.284 |  | 0.027 | 1.528 |  | -0.196 | 1.554 |  | 0.900 |
| Body weight | A VS. B | - | - |  | - | - |  | - | - |  | - |
|  | A VS. C | -1.386 | 0.205 |  | -1.478 | 0.472 |  | 0.092 | 0.515 |  | 0.857 |
|  | A VS. D | -1.708 | 0.118 |  | -2.412 | 1.523 |  | 0.704 | 0.817 |  | 0.354 |
|  | A VS. E | - | - |  | - | - |  | - | - |  | - |
|  | A VS. F | -2.719 | 0.165 |  | -1.961 | 0.800 |  | -0.757 | 0.817 |  | 0.354 |
|  | A VS. G | - | - |  | - | - |  | - | - |  | - |
|  | A VS. H | - | - |  | - | - |  | - | - |  | - |
|  | A VS. I | - | - |  | - | - |  | - | - |  | - |
|  | A VS. J | -1.854 | 0.193 |  | -1.521 | 0.889 |  | -0.332 | 0.910 |  | 0.715 |
|  | C VS. D | -0.309 | 0.214 |  | -0.320 | 0.418 |  | 0.010 | 0.471 |  | 0.982 |
|  | E VS. F | -0.700 | 0.165 |  | -1.458 | 0.801 |  | 0.757 | 0.817 |  | 0.354 |
|  | G VS. H | - | - |  | - | - |  | - | - |  | - |
|  | I VS. J | 0.062 | 0.191 |  | -0.270 | 0.890 |  | 0.332 | 0.910 |  | 0.715 |
| SBP | A VS. B | - | - |  | - | - |  | - | - |  | - |
|  | A VS. C | -4.270 | 1.463 |  | 0.458 | 3.137 |  | -4.729 | 3.365 |  | 0.160 |
|  | A VS. D | - | - |  | - | - |  | - | - |  | - |
|  | A VS. E | - | - |  | - | - |  | - | - |  | - |
|  | A VS. F | - | - |  | - | - |  | - | - |  | - |
|  | A VS. G | - | - |  | - | - |  | - | - |  | - |
|  | A VS. H | - | - |  | - | - |  | - | - |  | - |
|  | A VS. I | - | - |  | - | - |  | - | - |  | - |
|  | A VS. J | - | - |  | - | - |  | - | - |  | - |
|  | C VS. D | 0.091 | 0.148 |  | -0.245 | 0.487 |  | 0.335 | 0.510 |  | 0.511 |
|  | E VS. F | - | - |  | - | - |  | - | - |  | - |
|  | G VS. H | 0.225 | 0.165 |  | -0.236 | 0.739 |  | 0.462 | 0.757 |  | 0.541 |
|  | I VS. J | - | - |  | - | - |  | - | - |  | - |
| DBP | A VS. B | - | - |  | - | - |  | - | - |  | - |
|  | A VS. C | -0.773 | 0.935 |  | 1.709 | 3.894 |  | -2.483 | 3.975 |  | 0.532 |
|  | A VS. D | - | - |  | - | - |  | - | - |  | - |
|  | A VS. E | - | - |  | - | - |  | - | - |  | - |
|  | A VS. F | -2.156 | 0.339 |  | 0.066 | 2.134 |  | -2.134 | -2.223 |  | 0.304 |
|  | A VS. G | - | - |  | - | - |  | - | - |  | - |
|  | A VS. H | - | - |  | - | - |  | - | - |  | - |
|  | A VS. I | - | - |  | - | - |  | - | - |  | - |
|  | A VS. J | - | - |  | - | - |  | - | - |  | - |
|  | C VS. D | -1.367 | 1.069 |  | 1.115 | 3.789 |  | -2.483 | 3.975 |  | 0.532 |
|  | E VS. F | 0.194 | 0.328 |  | -2.028 | 2.139 |  | 2.223 | 2.165 |  | 0.304 |
|  | G VS. H | - | - |  | - | - |  | - | - |  | - |
|  | I VS. J | - | - |  | - | - |  | - | - |  | - |
| hypoglycemia | A VS. B | - | - |  | - | - |  | - | - |  | - |
|  | A VS. C | 0.122 | 0.554 |  | 0.027 | 1.016 |  | 0.095 | 1.150 |  | 0.934 |
|  | A VS. D | - | - |  | - | - |  | - | - |  | - |
|  | A VS. E | - | - |  | - | - |  | - | - |  | - |
|  | A VS. F | 0.469 | 0.682 |  | -0.437 | 1.581 |  | 0.906 | 1.762 |  | 0.607 |
|  | A VS. G | - | - |  | - | - |  | - | - |  | - |
|  | A VS. H | - | - |  | - | - |  | - | - |  | - |
|  | A VS. I | - | - |  | - | - |  | - | - |  | - |
|  | A VS. J | 0.230 | 0.280 |  | 3.818 | 3.208 |  | -3.587 | 3.222 |  | 0.266 |
|  | C VS. D | 0.225 | 0.527 |  | 0.129 | 1.060 |  | 0.095 | 1.150 |  | 0.934 |
|  | E VS. F | -0.220 | 0.586 |  | 0.686 | 1.692 |  | -0.906 | 1.762 |  | 0.607 |
|  | G VS. H | - | - |  | - | - |  | - | - |  | - |
|  | I VS. J | 0.041 | 0.265 |  | -3.546 | 3.212 |  | 3.587 | 3.222 |  | 0.266 |
| urinary tract infection | A VS. B | - | - |  | - | - |  | - | - |  | - |
|  | A VS. C | 0.408 | 0.315 |  | 0.383 | 0.603 |  | 0.024 | 0.696 |  | 0.972 |
|  | A VS. D | - | - |  | - | - |  | - | - |  | - |
|  | A VS. E | - | - |  | - | - |  | - | - |  | - |
|  | A VS. F | -0.170 | 0.186 |  | 2.192 | 3.298 |  | -2.362 | 3.303 |  | 0.475 |
|  | A VS. G | - | - |  | - | - |  | - | - |  | - |
|  | A VS. H | - | - |  | - | - |  | - | - |  | - |
|  | A VS. I | - | - |  | - | - |  | - | - |  | - |
|  | A VS. J | -0.225 | 0.082 |  | -4.744 | 3.009 |  | 4.518 | 3.010 |  | 0.133 |
|  | C VS. D | -0.063 | 0.291 |  | -0.087 | 0.637 |  | 0.024 | 0.696 |  | 0.972 |
|  | E VS. F | -0.046 | 0.189 |  | -2.408 | 3.298 |  | 2.362 | 3.303 |  | 0.475 |
|  | G VS. H | - | - |  | - | - |  | - | - |  | - |
|  | I VS. J | -0.193 | 0.082 |  | 4.325 | 3.009 |  | -4.518 | 3.010 |  | 0.133 |
| Genital mycotic infection | A VS. B | - | - |  | - | - |  | - | - |  | - |
|  | A VS. C | 0.319 | 0.677 |  | 2.784 | 1.654 |  | -2.465 | 1.931 |  | 0.202 |
|  | A VS. D | 1.064 | 0.338 |  | -1.243 | 2.404 |  | 2.307 | 2.440 |  | 0.344 |
|  | A VS. E |  |  |  |  |  |  |  |  |  |  |
|  | A VS. F | 1.074 | 0.286 |  | 0.674 | 3.303 |  | 0.4000 | 3.331 |  | 0.904 |
|  | A VS. G |  |  |  |  |  |  |  |  |  |  |
|  | A VS. H |  |  |  |  |  |  |  |  |  |  |
|  | A VS. I |  |  |  |  |  |  |  |  |  |  |
|  | A VS. J | 1.420 | 0.158 |  | -1.090 | 2.863 |  | 2.510 | 2.872 |  | 0.382 |
|  | C VS. D | -0.405 | 0.736 |  | 1.273 | 0.985 |  | -1.678 | 1.265 |  | 0.185 |
|  | E VS. F | -0.182 | 0.176 |  | 0.217 | 3.327 |  | -0.400 | 3.331 |  | 0.904 |
|  | G VS. H |  |  |  |  |  |  |  |  |  |  |
|  | I VS. J | 0.183 | 0.105 |  | 2.694 | 2.870 |  | -2.510 | 2.872 |  | 0.382 |

**Table S5 SUCRAs of all interventions according to HbA1c , FPG, Body weight, SBP and DBP**

| Interventions | HbA1c | FPG | Body weight | SBP | DBP |
| --- | --- | --- | --- | --- | --- |
| Placebo | 0 | 0 | 0 | 0.2 | 3.3 |
| Bexagliflozin 20mg | 24.2 | 18.7 | 80.1 | 50.7 | 72.8 |
| Dapagliflozin 5mg | 25.3 | 43.4 | 14.2 | 58.8 | 23.4 |
| Dapagliflozin 10mg | 32.6 | 27.0 | 36.9 | 50.0 | 59.7 |
| Canagliflozin 100 mg | 75.8 | 62.3 | 66.9 | 60.1 | 76.5 |
| Canagliflozin 300 mg | 96.9 | 86.9 | 99.8 | 68.3 | 69.8 |
| Empagliflozin 10mg | 64.0 | 68.9 | 36.1 | 37.3 | 18.2 |
| Empagliflozin 25mg | 83.4 | 85.7 | 55.6 | 65.9 | 41.4 |
| Ertugliflozin 5 mg | 44.5 | 46.0 | 58.6 | 44.9 | 57.0 |
| Ertugliflozin 15 mg | 53.3 | 61.1 | 52.0 | 63.7 | 77.9 |

HbA1c: glycosylated haemoglobin; FPG: fasting plasma glucose; SBP: systolic blood pressure; DBP: diastolic blood pressure.

# Figure S1 Network plots for four outcomes.

A B


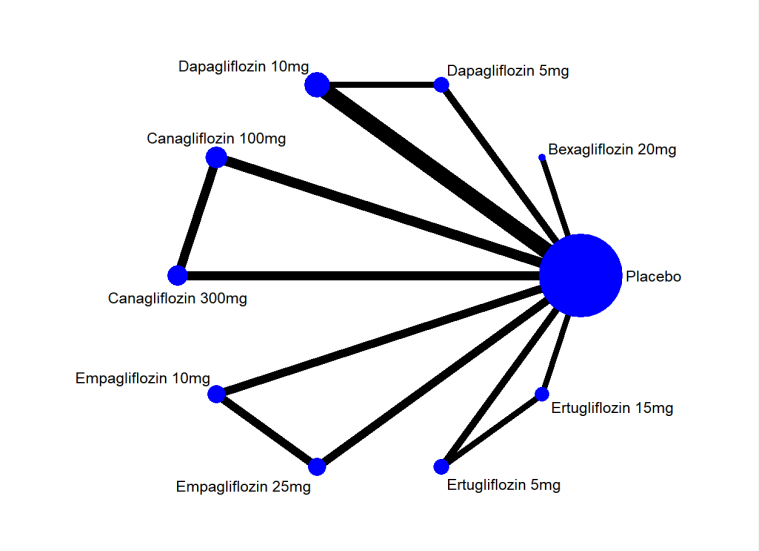

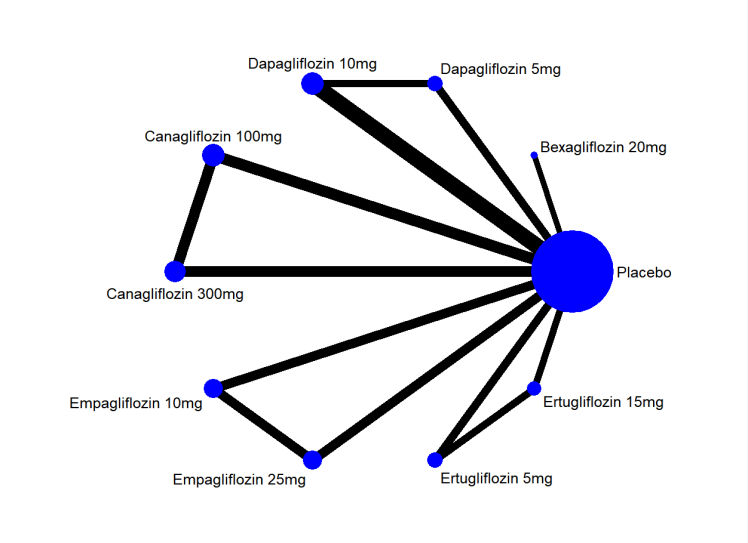


C D


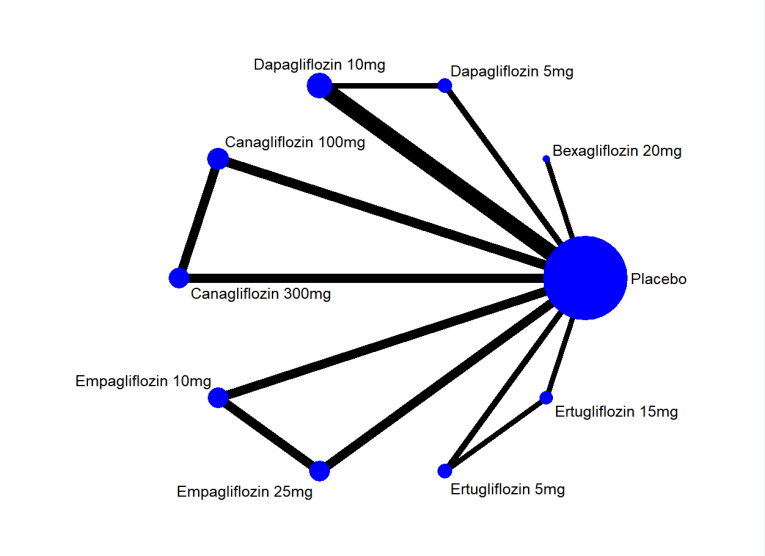

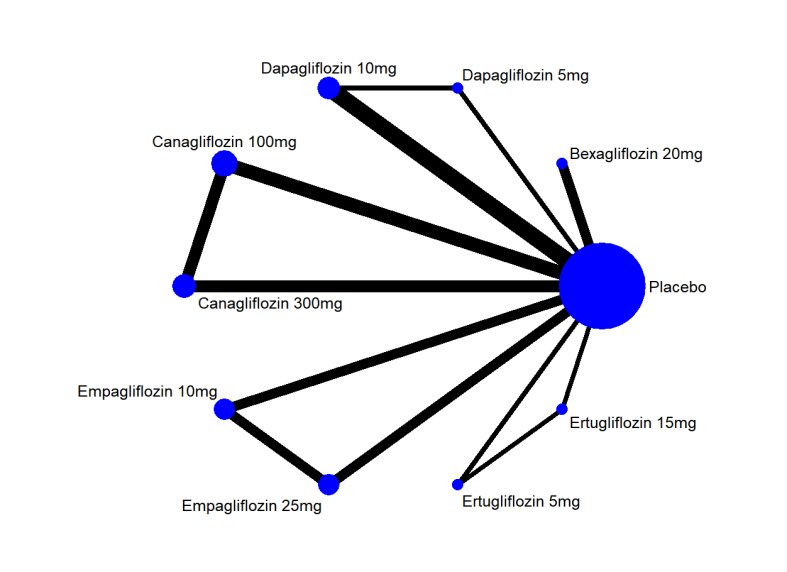


E F


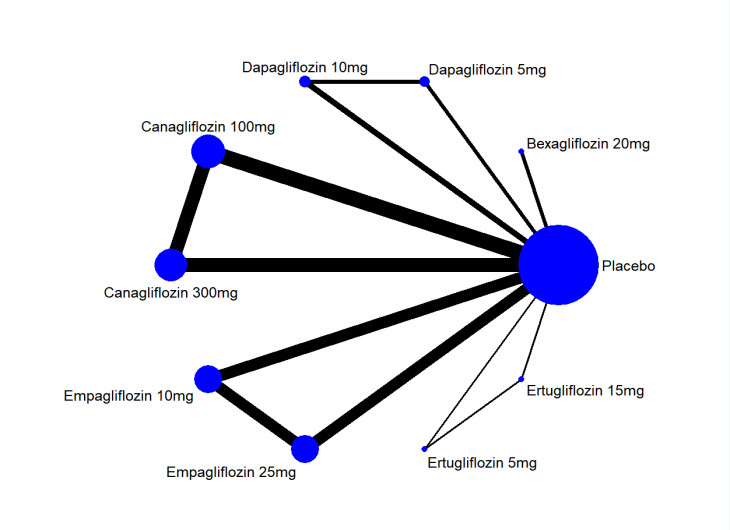

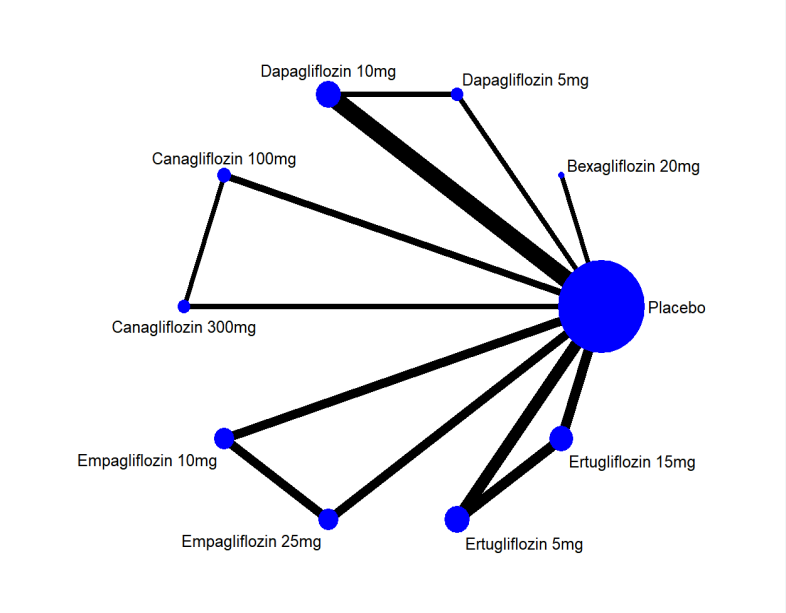


G H


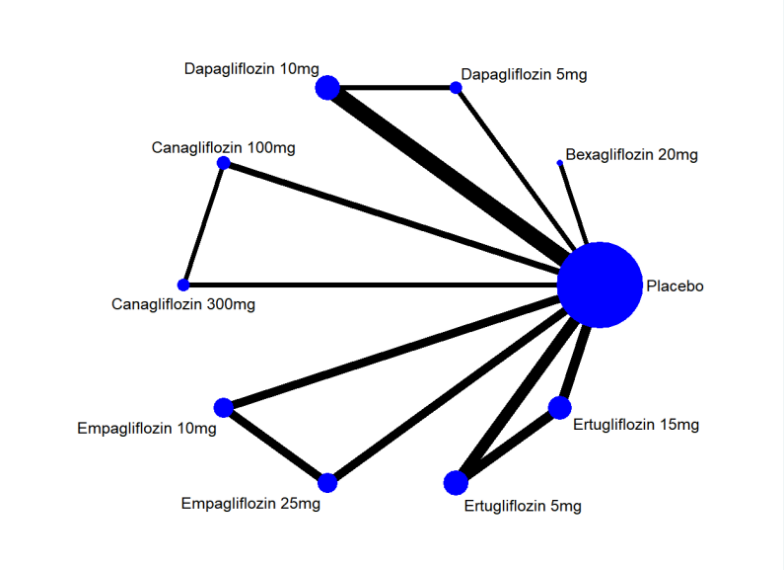

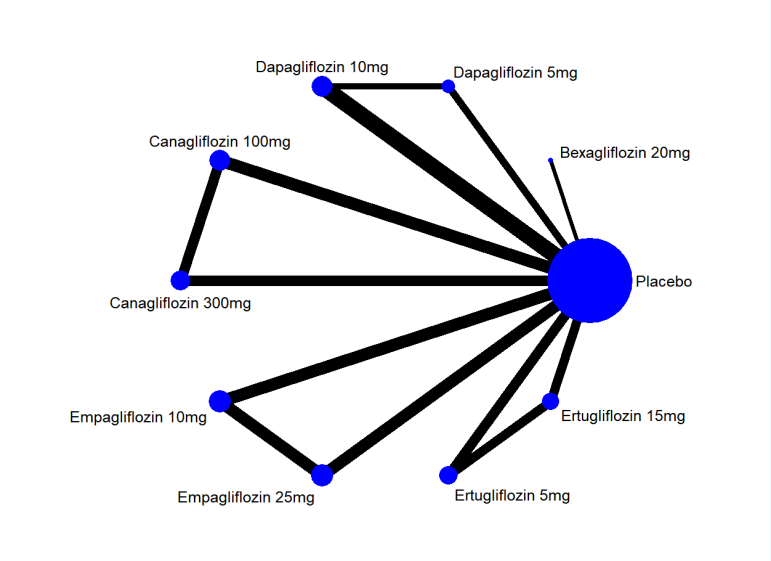


(A)HbA1c; (B)FPG; (C)Body weight; (D)SBP; (E)DBP; (F) Hypoglycemia ;(G)Urinary tract infection;(H)Genital mycotic infection. The magnitudes of the nodes are weighted based on the sample volume of interventions, and the thicknesses of the lines are weighted according to the count of included studies. HbA1c: glycosylated haemoglobin; FPG: fasting plasma glucose; SBP: systolic blood pressure; DBP: diastolic blood pressure.

**Figure S2: The results of network meta-analysis for body weight.**


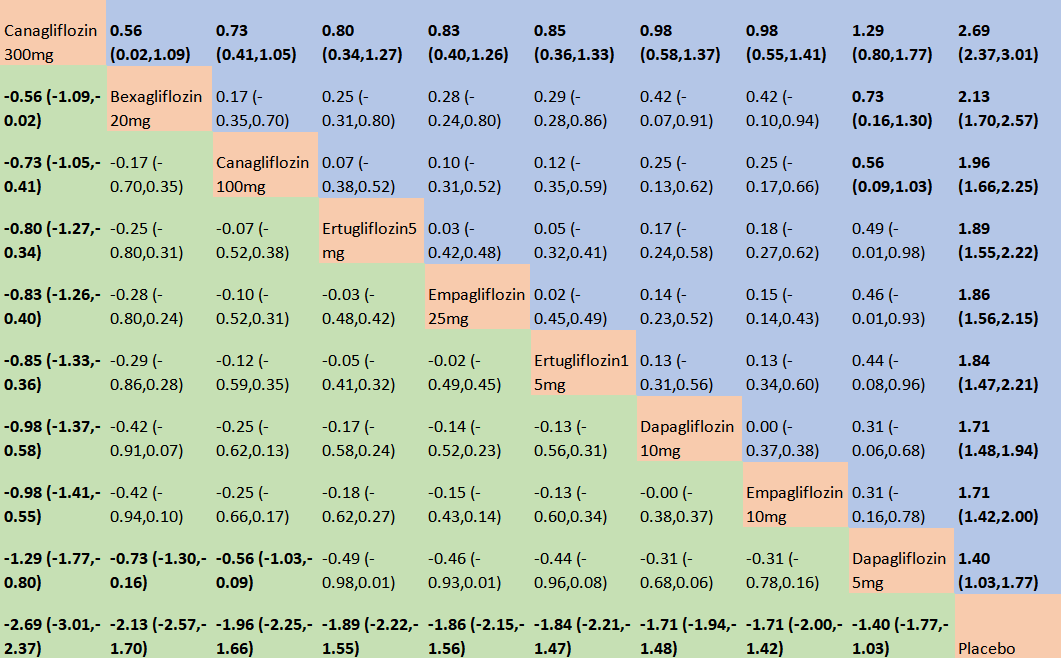


The central block of interventions divides the graph into upper and lower triangular sections. In the lower triangle, efficacy estimates represent the ratio of column-defined to row-defined treatments: a MD > 0 favors the column treatment, while a MD < 0 favors the row treatment. The upper triangle is the symmetrical mirror of the lower one. Statistically significant results are in bold.

**Figure S3: The results of network meta-analysis for SBP.**


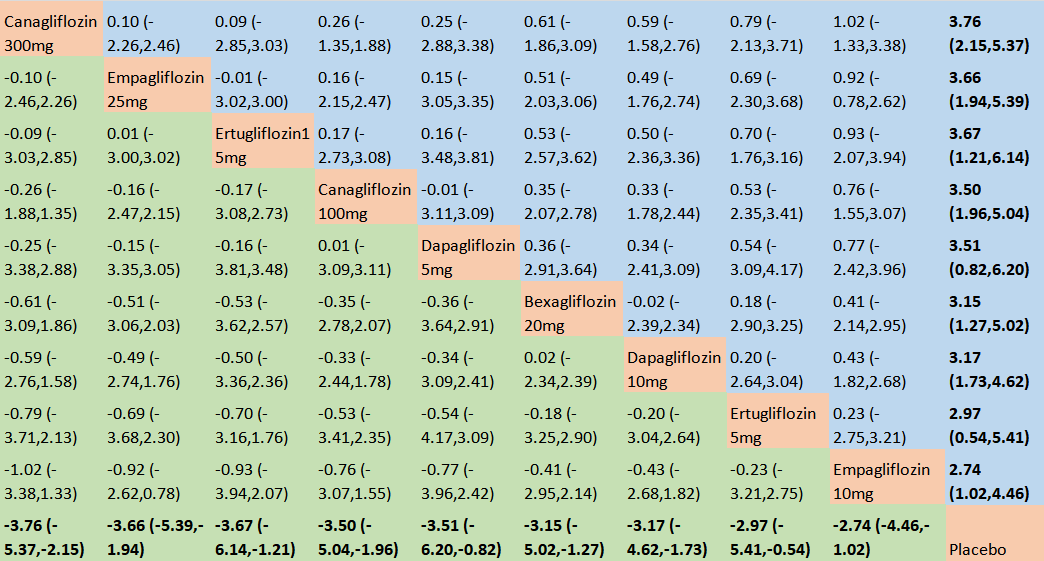
 The central block of interventions divides the graph into upper and lower triangular sections. In the lower triangle, efficacy estimates represent the ratio of column-defined to row-defined treatments: a MD > 0 favors the column treatment, while a MD < 0 favors the row treatment. The upper triangle is the symmetrical mirror of the lower one. Statistically significant results are in bold.

**Figure S4: The results of network meta-analysis for DBP.**


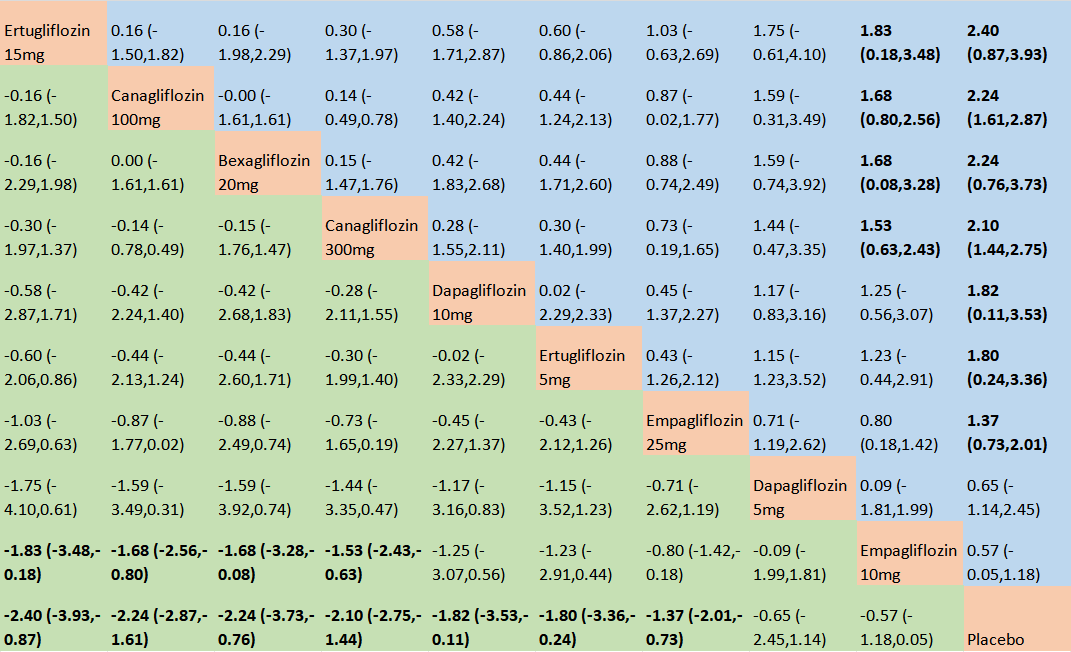


The central block of interventions divides the graph into upper and lower triangular sections. In the lower triangle, efficacy estimates represent the ratio of column-defined to row-defined treatments: a MD > 0 favors the column treatment, while a MD < 0 favors the row treatment. The upper triangle is the symmetrical mirror of the lower one. Statistically significant results are in bold.

**Figure S5: The results of network meta-analysis for hypoglycemia.**


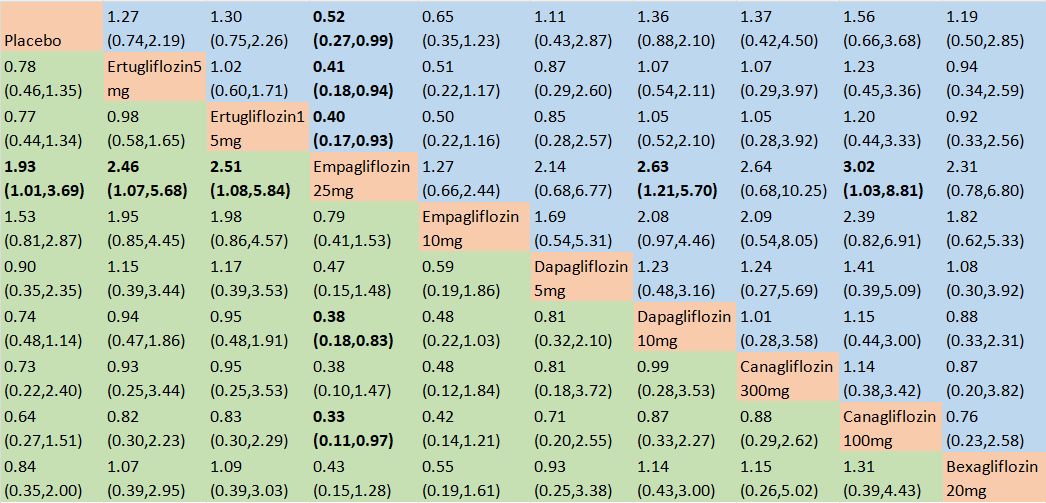


The central block of interventions divides the graph into upper and lower triangular sections. In the lower triangle, efficacy estimates represent the ratio of column-defined to row-defined treatments: a OR > 1 favors the column treatment, while a OR < 1 favors the row treatment. The upper triangle is the symmetrical mirror of the lower one. Statistically significant results are in bold.

**Figure S6: The results of network meta-analysis for urinary tract infection.**


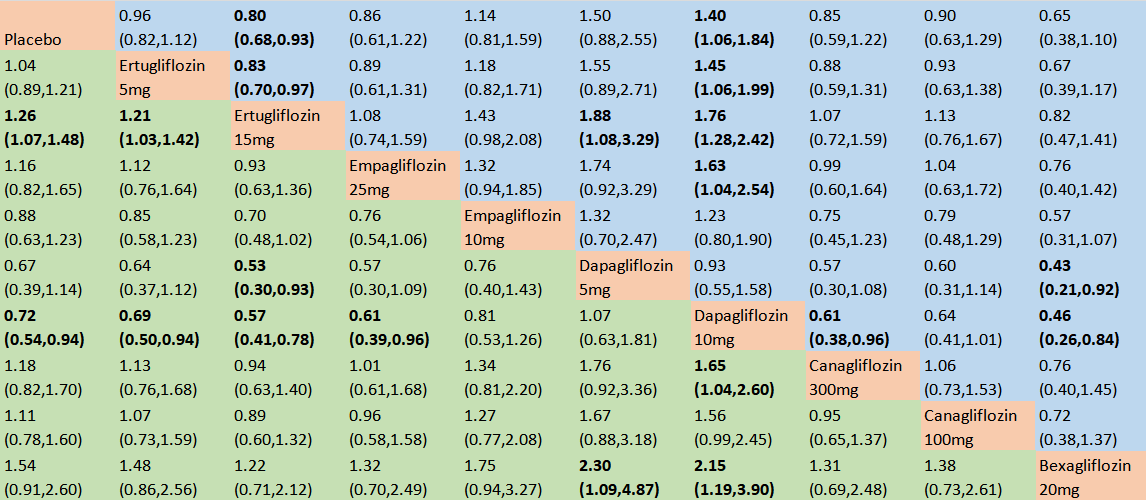


The central block of interventions divides the graph into upper and lower triangular sections. In the lower triangle, efficacy estimates represent the ratio of column-defined to row-defined treatments: a OR > 1 favors the column treatment, while a OR < 1 favors the row treatment. The upper triangle is the symmetrical mirror of the lower one. Statistically significant results are in bold.

**Figure S7: The results of network meta-analysis for genital mycotic infection.**


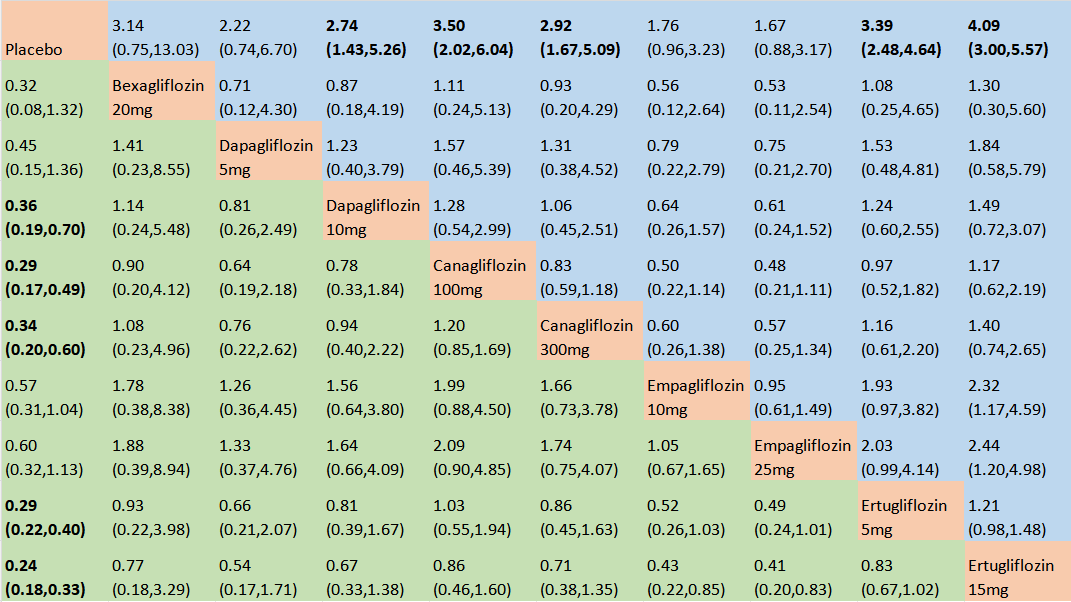


The central block of interventions divides the graph into upper and lower triangular sections. In the lower triangle, efficacy estimates represent the ratio of column-defined to row-defined treatments: a OR >1 favors the column treatment, while a OR < 1 favors the row treatment. The upper triangle is the symmetrical mirror of the lower one. Statistically significant results are in bold.

**Table S6: CINeMA ratings for all comparisons of the four outcomes**

| **Comparison** | **Nature of evidence** | **Confidence level** | **Downgrading** |
| --- | --- | --- | --- |
| **HbA1c** | | | |
| Bexagliflozin20mg:PLACEBO | mixed | High |  |
| Canagliflozin100mg:Canagliflozin300mg | mixed | Very low | Imprecision、Heterogeneity、Serious Incoherence |
| Canagliflozin100mg:PLACEBO | mixed | High |  |
| Canagliflozin300mg:PLACEBO | mixed | High |  |
| Dapagliflozin10mg:Dapagliflozin5mg | mixed | Low | Serious Imprecision |
| Dapagliflozin10mg:PLACEBO | mixed | High |  |
| Dapagliflozin5mg:PLACEBO | mixed | High |  |
| Empagliflozin10mg:Empagliflozin25mg | mixed | Low | Imprecision、Heterogeneity |
| Empagliflozin10mg:PLACEBO | mixed | High |  |
| Empagliflozin25mg:PLACEBO | mixed | High |  |
| Ertugliflozin15mg:Ertugliflozin5mg | mixed | Low | Serious Imprecision |
| Ertugliflozin15mg:PLACEBO | mixed | High |  |
| Ertugliflozin5mg:PLACEBO | mixed | High |  |
| Bexagliflozin20mg:Canagliflozin100mg | indrect | Low | Serious Heterogeneity |
| Bexagliflozin20mg:Canagliflozin300mg | indrect | Moderate | Heterogeneity |
| Bexagliflozin20mg:Dapagliflozin10mg | indrect | Low | Serious Imprecision |
| Bexagliflozin20mg:Dapagliflozin5mg | indrect | Low | Serious Imprecision |
| Bexagliflozin20mg:Empagliflozin10mg | indrect | Low | Imprecision、Heterogeneity |
| Bexagliflozin20mg:Empagliflozin25mg | indrect | Low | Serious Heterogeneity |
| Bexagliflozin20mg:Ertugliflozin15mg | indrect | Low | Imprecision、Heterogeneity |
| Bexagliflozin20mg:Ertugliflozin5mg | indrect | Low | Serious Imprecision |
| Canagliflozin100mg:Dapagliflozin10mg | indrect | Low | Serious Heterogeneity |
| Canagliflozin100mg:Dapagliflozin5mg | indrect | Low | Serious Heterogeneity |
| Canagliflozin100mg:Empagliflozin10mg | indrect | Low | Serious Imprecision |
| Canagliflozin100mg:Empagliflozin25mg | indrect | Low | Serious Imprecision |
| Canagliflozin100mg:Ertugliflozin15mg | indrect | Low | Imprecision、Heterogeneity |
| Canagliflozin100mg:Ertugliflozin5mg | indrect | Low | Imprecision、Heterogeneity |
| Canagliflozin300mg:Dapagliflozin10mg | indrect | Moderate | Heterogeneity |
| Canagliflozin300mg:Dapagliflozin5mg | indrect | Moderate | Heterogeneity |
| Canagliflozin300mg:Empagliflozin10mg | indrect | Low | Imprecision、Heterogeneity |
| Canagliflozin300mg:Empagliflozin25mg | indrect | Low | Imprecision、Heterogeneity |
| Canagliflozin300mg:Ertugliflozin15mg | indrect | Low | Serious Heterogeneity |
| Canagliflozin300mg:Ertugliflozin5mg | indrect | Low | Serious Heterogeneity |
| Dapagliflozin10mg:Empagliflozin10mg | indrect | Low | Imprecision、Heterogeneity |
| Dapagliflozin10mg:Empagliflozin25mg | indrect | Low | Serious Heterogeneity |
| Dapagliflozin10mg:Ertugliflozin15mg | indrect | Low | Imprecision、Heterogeneity |
| Dapagliflozin10mg:Ertugliflozin5mg | indrect | Low | Serious Imprecision |
| Dapagliflozin5mg:Empagliflozin10mg | indrect | Low | Imprecision、Heterogeneity |
| Dapagliflozin5mg:Empagliflozin25mg | indrect | Low | Serious Heterogeneity |
| Dapagliflozin5mg:Ertugliflozin15mg | indrect | Low | Imprecision、Heterogeneity |
| Dapagliflozin5mg:Ertugliflozin5mg | indrect | Low | Serious Imprecision |
| Empagliflozin10mg:Ertugliflozin15mg | indrect | Low | Serious Imprecision |
| Empagliflozin10mg:Ertugliflozin5mg | indrect | Low | Serious Imprecision |
| Empagliflozin25mg:Ertugliflozin15mg | indrect | Low | Imprecision、Heterogeneity |
| Empagliflozin25mg:Ertugliflozin5mg | indrect | Low | Imprecision、Heterogeneity |
| **FPG** | | | |
| Bexagliflozin20mg:PLACEBO | mixed | Low | Serious Heterogeneity |
| Canagliflozin100mg:Canagliflozin300mg | mixed | Low | Serious Imprecision |
| Canagliflozin100mg:PLACEBO | mixed | High |  |
| Canagliflozin300mg:PLACEBO | mixed | High |  |
| Dapagliflozin10mg:Dapagliflozin5mg | mixed | Low | Serious Imprecision |
| Dapagliflozin10mg:PLACEBO | mixed | Low | Serious Heterogeneity |
| Dapagliflozin5mg:PLACEBO | mixed | Low | Serious Heterogeneity |
| Empagliflozin10mg:Empagliflozin25mg | mixed | Low | Serious Imprecision |
| Empagliflozin10mg:PLACEBO | mixed | High |  |
| Empagliflozin25mg:PLACEBO | mixed | High |  |
| Ertugliflozin15mg:Ertugliflozin5mg | mixed | Low | Serious Imprecision |
| Ertugliflozin15mg:PLACEBO | mixed | High |  |
| Ertugliflozin5mg:PLACEBO | mixed | Low | Serious Heterogeneity |
| Bexagliflozin20mg:Canagliflozin100mg | indrect | Low | Serious Imprecision |
| Bexagliflozin20mg:Canagliflozin300mg | indrect | Low | Serious Heterogeneity |
| Bexagliflozin20mg:Dapagliflozin10mg | indrect | Low | Serious Imprecision |
| Bexagliflozin20mg:Dapagliflozin5mg | indrect | Low | Serious Imprecision |
| Bexagliflozin20mg:Empagliflozin10mg | indrect | Low | Imprecision、Heterogeneity |
| Bexagliflozin20mg:Empagliflozin25mg | indrect | Low | Serious Heterogeneity |
| Bexagliflozin20mg:Ertugliflozin15mg | indrect | Low | Serious Imprecision |
| Bexagliflozin20mg:Ertugliflozin5mg | indrect | Low | Serious Imprecision |
| Canagliflozin100mg:Dapagliflozin10mg | indrect | Low | Serious Imprecision |
| Canagliflozin100mg:Dapagliflozin5mg | indrect | Low | Serious Imprecision |
| Canagliflozin100mg:Empagliflozin10mg | indrect | Low | Serious Imprecision |
| Canagliflozin100mg:Empagliflozin25mg | indrect | Low | Serious Imprecision |
| Canagliflozin100mg:Ertugliflozin15mg | indrect | Low | Serious Imprecision |
| Canagliflozin100mg:Ertugliflozin5mg | indrect | Low | Serious Imprecision |
| Canagliflozin300mg:Dapagliflozin10mg | indrect | Low | Serious Heterogeneity |
| Canagliflozin300mg:Dapagliflozin5mg | indrect | Low | Serious Imprecision |
| Canagliflozin300mg:Empagliflozin10mg | indrect | Low | Serious Imprecision |
| Canagliflozin300mg:Empagliflozin25mg | indrect | Low | Serious Imprecision |
| Canagliflozin300mg:Ertugliflozin15mg | indrect | Low | Serious Imprecision |
| Canagliflozin300mg:Ertugliflozin5mg | indrect | Low | Serious Imprecision |
| Dapagliflozin10mg:Empagliflozin10mg | indrect | Low | Serious Imprecision |
| Dapagliflozin10mg:Empagliflozin25mg | indrect | Low | Serious Heterogeneity |
| Dapagliflozin10mg:Ertugliflozin15mg | indrect | Low | Serious Imprecision |
| Dapagliflozin10mg:Ertugliflozin5mg | indrect | Low | Serious Imprecision |
| Dapagliflozin5mg:Empagliflozin10mg | indrect | Low | Serious Imprecision |
| Dapagliflozin5mg:Empagliflozin25mg | indrect | Low | Serious Imprecision |
| Dapagliflozin5mg:Ertugliflozin15mg | indrect | Low | Serious Imprecision |
| Dapagliflozin5mg:Ertugliflozin5mg | indrect | Low | Serious Imprecision |
| Empagliflozin10mg:Ertugliflozin15mg | indrect | Low | Serious Imprecision |
| Empagliflozin10mg:Ertugliflozin5mg | indrect | Low | Serious Imprecision |
| Empagliflozin25mg:Ertugliflozin15mg | indrect | Low | Serious Imprecision |
| Empagliflozin25mg:Ertugliflozin5mg | indrect | Low | Serious Imprecision |
| **Body weight** | | | |
| Bexagliflozin20mg:PLACEBO | mixed | High |  |
| Canagliflozin100mg:Canagliflozin300mg | mixed | High |  |
| Canagliflozin100mg:PLACEBO | mixed | High |  |
| Canagliflozin300mg:PLACEBO | mixed | High |  |
| Dapagliflozin10mg:Dapagliflozin5mg | mixed | Low | Imprecision、Heterogeneity |
| Dapagliflozin10mg:PLACEBO | mixed | High |  |
| Dapagliflozin5mg:PLACEBO | mixed | High |  |
| Empagliflozin10mg:Empagliflozin25mg | mixed | Low | Serious Imprecision |
| Empagliflozin10mg:PLACEBO | mixed | High |  |
| Empagliflozin25mg:PLACEBO | mixed | High |  |
| Ertugliflozin15mg:Ertugliflozin5mg | mixed | Low | Serious Imprecision |
| Ertugliflozin15mg:PLACEBO | mixed | High |  |
| Ertugliflozin5mg:PLACEBO | mixed | High |  |
| Bexagliflozin20mg:Canagliflozin100mg | indrect | Low | Serious Imprecision |
| Bexagliflozin20mg:Canagliflozin300mg | indrect | Low |  |
| Bexagliflozin20mg:Dapagliflozin10mg | indrect | Low | Imprecision、Heterogeneity |
| Bexagliflozin20mg:Dapagliflozin5mg | indrect | Moderate | Heterogeneity |
| Bexagliflozin20mg:Empagliflozin10mg | indrect | Low | Imprecision、Heterogeneity |
| Bexagliflozin20mg:Empagliflozin25mg | indrect | Low | Serious Imprecision |
| Bexagliflozin20mg:Ertugliflozin15mg | indrect | Low | Serious Imprecision |
| Bexagliflozin20mg:Ertugliflozin5mg | indrect | Low | Serious Imprecision |
| Canagliflozin100mg:Dapagliflozin10mg | indrect | Low | Serious Imprecision |
| Canagliflozin100mg:Dapagliflozin5mg | indrect | Low | Serious Heterogeneity |
| Canagliflozin100mg:Empagliflozin10mg | indrect | Low | Serious Imprecision |
| Canagliflozin100mg:Empagliflozin25mg | indrect | Low | Serious Imprecision |
| Canagliflozin100mg:Ertugliflozin15mg | indrect | Low | Serious Imprecision |
| Canagliflozin100mg:Ertugliflozin5mg | indrect | Low | Serious Imprecision |
| Canagliflozin300mg:Dapagliflozin10mg | indrect | High |  |
| Canagliflozin300mg:Dapagliflozin5mg | indrect | High |  |
| Canagliflozin300mg:Empagliflozin10mg | indrect | High |  |
| Canagliflozin300mg:Empagliflozin25mg | indrect | High |  |
| Canagliflozin300mg:Ertugliflozin15mg | indrect | High |  |
| Canagliflozin300mg:Ertugliflozin5mg | indrect | High |  |
| Dapagliflozin10mg:Empagliflozin10mg | indrect | Low | Serious Imprecision |
| Dapagliflozin10mg:Empagliflozin25mg | indrect | Low | Serious Imprecision |
| Dapagliflozin10mg:Ertugliflozin15mg | indrect | Low | Serious Imprecision |
| Dapagliflozin10mg:Ertugliflozin5mg | indrect | Low | Serious Imprecision |
| Dapagliflozin5mg:Empagliflozin10mg | indrect | Low | Serious Imprecision |
| Dapagliflozin5mg:Empagliflozin25mg | indrect | Low | Imprecision、Heterogeneity |
| Dapagliflozin5mg:Ertugliflozin15mg | indrect | Low | Imprecision、Heterogeneity |
| Dapagliflozin5mg:Ertugliflozin5mg | indrect | Low | Imprecision、Heterogeneity |
| Empagliflozin10mg:Ertugliflozin15mg | indrect | Low | Serious Imprecision |
| Empagliflozin10mg:Ertugliflozin5mg | indrect | Low | Serious Imprecision |
| Empagliflozin25mg:Ertugliflozin15mg | indrect | Low | Serious Imprecision |
| Empagliflozin25mg:Ertugliflozin5mg | indrect | Low | Serious Imprecision |
| **SBP** | | | |
| Bexagliflozin20mg:PLACEBO | mixed | Low | Serious Heterogeneity |
| Canagliflozin100mg:Canagliflozin300mg | mixed | Low | Serious Imprecision |
| Canagliflozin100mg:PLACEBO | mixed | High |  |
| Canagliflozin300mg:PLACEBO | mixed | High |  |
| Dapagliflozin10mg:Dapagliflozin5mg | mixed | Low | Serious Imprecision |
| Dapagliflozin10mg:PLACEBO | mixed | Low | Serious Heterogeneity |
| Dapagliflozin5mg:PLACEBO | mixed | Low | Serious Heterogeneity |
| Empagliflozin10mg:Empagliflozin25mg | mixed | Low | Serious Imprecision |
| Empagliflozin10mg:PLACEBO | mixed | Low | Serious Heterogeneity |
| Empagliflozin25mg:PLACEBO | mixed | High |  |
| Ertugliflozin15mg:Ertugliflozin5mg | mixed | Low | Serious Imprecision |
| Ertugliflozin15mg:PLACEBO | mixed | Low | Serious Heterogeneity |
| Ertugliflozin5mg:PLACEBO | mixed | Low | Serious Heterogeneity |
| Bexagliflozin20mg:Canagliflozin100mg | indrect | Low | Serious Imprecision |
| Bexagliflozin20mg:Canagliflozin300mg | indrect | Low | Serious Imprecision |
| Bexagliflozin20mg:Dapagliflozin10mg | indrect | Low | Serious Imprecision |
| Bexagliflozin20mg:Dapagliflozin5mg | indrect | Low | Serious Imprecision |
| Bexagliflozin20mg:Empagliflozin10mg | indrect | Low | Serious Imprecision |
| Bexagliflozin20mg:Empagliflozin25mg | indrect | Low | Serious Imprecision |
| Bexagliflozin20mg:Ertugliflozin15mg | indrect | Low | Serious Imprecision |
| Bexagliflozin20mg:Ertugliflozin5mg | indrect | Low | Serious Imprecision |
| Canagliflozin100mg:Dapagliflozin10mg | indrect | Low | Serious Imprecision |
| Canagliflozin100mg:Dapagliflozin5mg | indrect | Low | Serious Imprecision |
| Canagliflozin100mg:Empagliflozin10mg | indrect | Low | Serious Imprecision |
| Canagliflozin100mg:Empagliflozin25mg | indrect | Low | Serious Imprecision |
| Canagliflozin100mg:Ertugliflozin15mg | indrect | Low | Serious Imprecision |
| Canagliflozin100mg:Ertugliflozin5mg | indrect | Low | Serious Imprecision |
| Canagliflozin300mg:Dapagliflozin10mg | indrect | Low | Serious Imprecision |
| Canagliflozin300mg:Dapagliflozin5mg | indrect | Low | Serious Imprecision |
| Canagliflozin300mg:Empagliflozin10mg | indrect | Low | Serious Imprecision |
| Canagliflozin300mg:Empagliflozin25mg | indrect | Low | Serious Imprecision |
| Canagliflozin300mg:Ertugliflozin15mg | indrect | Low | Serious Imprecision |
| Canagliflozin300mg:Ertugliflozin5mg | indrect | Low | Serious Imprecision |
| Dapagliflozin10mg:Empagliflozin10mg | indrect | Low | Serious Imprecision |
| Dapagliflozin10mg:Empagliflozin25mg | indrect | Low | Serious Imprecision |
| Dapagliflozin10mg:Ertugliflozin15mg | indrect | Low | Serious Imprecision |
| Dapagliflozin10mg:Ertugliflozin5mg | indrect | Low | Serious Imprecision |
| Dapagliflozin5mg:Empagliflozin10mg | indrect | Low | Serious Imprecision |
| Dapagliflozin5mg:Empagliflozin25mg | indrect | Low | Serious Imprecision |
| Dapagliflozin5mg:Ertugliflozin15mg | indrect | Low | Serious Imprecision |
| Dapagliflozin5mg:Ertugliflozin5mg | indrect | Low | Serious Imprecision |
| Empagliflozin10mg:Ertugliflozin15mg | indrect | Low | Serious Imprecision |
| Empagliflozin10mg:Ertugliflozin5mg | indrect | Low | Serious Imprecision |
| Empagliflozin25mg:Ertugliflozin15mg | indrect | Low | Serious Imprecision |
| Empagliflozin25mg:Ertugliflozin5mg | indrect | Low | Serious Imprecision |
| **DBP** | | | |
| Bexagliflozin20mg:PLACEBO | mixed | High |  |
| Canagliflozin100mg:Canagliflozin300mg | mixed | Low | Serious Imprecision |
| Canagliflozin100mg:PLACEBO | mixed | High |  |
| Canagliflozin300mg:PLACEBO | mixed | High |  |
| Dapagliflozin10mg:Dapagliflozin5mg | mixed | Low | Serious Imprecision |
| Dapagliflozin10mg:PLACEBO | mixed | Low | Serious Heterogeneity |
| Dapagliflozin5mg:PLACEBO | mixed | Low | Serious Imprecision |
| Empagliflozin10mg:Empagliflozin25mg | mixed | Low | Serious Imprecision |
| Empagliflozin10mg:PLACEBO | mixed | Low | Serious Incoherence |
| Empagliflozin25mg:PLACEBO | mixed | High |  |
| Ertugliflozin15mg:Ertugliflozin5mg | mixed | Low | Serious Imprecision |
| Ertugliflozin15mg:PLACEBO | mixed | High |  |
| Ertugliflozin5mg:PLACEBO | mixed | Low | Serious Heterogeneity |
| Bexagliflozin20mg:Canagliflozin100mg | indrect | Low | Serious Imprecision |
| Bexagliflozin20mg:Canagliflozin300mg | indrect | Low | Serious Imprecision |
| Bexagliflozin20mg:Dapagliflozin10mg | indrect | Low | Serious Imprecision |
| Bexagliflozin20mg:Dapagliflozin5mg | indrect | Low | Serious Imprecision |
| Bexagliflozin20mg:Empagliflozin10mg | indrect | Low | Serious Imprecision |
| Bexagliflozin20mg:Empagliflozin25mg | indrect | Low | Serious Imprecision |
| Bexagliflozin20mg:Ertugliflozin15mg | indrect | Low | Serious Imprecision |
| Bexagliflozin20mg:Ertugliflozin5mg | indrect | Low | Serious Imprecision |
| Canagliflozin100mg:Dapagliflozin10mg | indrect | Low | Serious Imprecision |
| Canagliflozin100mg:Dapagliflozin5mg | indrect | Low | Serious Imprecision |
| Canagliflozin100mg:Empagliflozin10mg | indrect | High |  |
| Canagliflozin100mg:Empagliflozin25mg | indrect | Low | Imprecision、Heterogeneity |
| Canagliflozin100mg:Ertugliflozin15mg | indrect | Low | Serious Imprecision |
| Canagliflozin100mg:Ertugliflozin5mg | indrect | Low | Serious Imprecision |
| Canagliflozin300mg:Dapagliflozin10mg | indrect | Low | Serious Imprecision |
| Canagliflozin300mg:Dapagliflozin5mg | indrect | Low | Serious Imprecision |
| Canagliflozin300mg:Empagliflozin10mg | indrect | Low | Serious Heterogeneity |
| Canagliflozin300mg:Empagliflozin25mg | indrect | Low | Serious Imprecision |
| Canagliflozin300mg:Ertugliflozin15mg | indrect | Low | Serious Imprecision |
| Canagliflozin300mg:Ertugliflozin5mg | indrect | Low | Serious Imprecision |
| Dapagliflozin10mg:Empagliflozin10mg | indrect | Low | Serious Imprecision |
| Dapagliflozin10mg:Empagliflozin25mg | indrect | Low | Serious Imprecision |
| Dapagliflozin10mg:Ertugliflozin15mg | indrect | Low | Serious Imprecision |
| Dapagliflozin10mg:Ertugliflozin5mg | indrect | Low | Serious Imprecision |
| Dapagliflozin5mg:Empagliflozin10mg | indrect | Low | Serious Imprecision |
| Dapagliflozin5mg:Empagliflozin25mg | indrect | Low | Serious Imprecision |
| Dapagliflozin5mg:Ertugliflozin15mg | indrect | Low | Serious Imprecision |
| Dapagliflozin5mg:Ertugliflozin5mg | indrect | Low | Serious Imprecision |
| Empagliflozin10mg:Ertugliflozin15mg | indrect | Low | Serious Imprecision |
| Empagliflozin10mg:Ertugliflozin5mg | indrect | Low | Serious Imprecision |
| Empagliflozin25mg:Ertugliflozin15mg | indrect | Low | Serious Imprecision |
| Empagliflozin25mg:Ertugliflozin5mg | indrect | Low | Serious Imprecision |
| **Hypoglycemia** | | | |
| Bexagliflozin20mg:PLACEBO | mixed | Low | Serious Imprecision |
| Canagliflozin100mg:Canagliflozin300mg | mixed | Low | Serious Imprecision |
| Canagliflozin100mg:PLACEBO | mixed | Low | Serious Imprecision |
| Canagliflozin300mg:PLACEBO | mixed | Low | Serious Imprecision |
| Dapagliflozin10mg:Dapagliflozin5mg | mixed | Low | Serious Imprecision |
| Dapagliflozin10mg:PLACEBO | mixed | Low | Imprecision、Heterogeneity |
| Dapagliflozin5mg:PLACEBO | mixed | Low | Serious Imprecision |
| Empagliflozin10mg:Empagliflozin25mg | mixed | Very low | Serious Imprecision、Serious Heterogeneity |
| Empagliflozin10mg:PLACEBO | mixed | Very low | Imprecision、Heterogeneity、Incoherence |
| Empagliflozin25mg:PLACEBO | mixed | Low | Serious Heterogeneity |
| Ertugliflozin15mg:Ertugliflozin5mg | mixed | Low | Serious Imprecision |
| Ertugliflozin15mg:PLACEBO | mixed | Low | Serious Imprecision |
| Ertugliflozin5mg:PLACEBO | mixed | Low | Serious Imprecision |
| Bexagliflozin20mg:Canagliflozin100mg | indrect | Low | Serious Imprecision |
| Bexagliflozin20mg:Canagliflozin300mg | indrect | Low | Serious Imprecision |
| Bexagliflozin20mg:Dapagliflozin10mg | indrect | Low | Serious Imprecision |
| Bexagliflozin20mg:Dapagliflozin5mg | indrect | Low | Serious Imprecision |
| Bexagliflozin20mg:Empagliflozin10mg | indrect | Low | Serious Imprecision |
| Bexagliflozin20mg:Empagliflozin25mg | indrect | Low | Imprecision、Heterogeneity |
| Bexagliflozin20mg:Ertugliflozin15mg | indrect | Low | Serious Imprecision |
| Bexagliflozin20mg:Ertugliflozin5mg | indrect | Low | Serious Imprecision |
| Canagliflozin100mg:Dapagliflozin10mg | indrect | Low | Serious Imprecision |
| Canagliflozin100mg:Dapagliflozin5mg | indrect | Low | Serious Imprecision |
| Canagliflozin100mg:Empagliflozin10mg | indrect | Low | Imprecision、Heterogeneity |
| Canagliflozin100mg:Empagliflozin25mg | indrect | Moderate | Heterogeneity |
| Canagliflozin100mg:Ertugliflozin15mg | indrect | Low | Serious Imprecision |
| Canagliflozin100mg:Ertugliflozin5mg | indrect | Low | Serious Imprecision |
| Canagliflozin300mg:Dapagliflozin10mg | indrect | Low | Serious Imprecision |
| Canagliflozin300mg:Dapagliflozin5mg | indrect | Low | Serious Imprecision |
| Canagliflozin300mg:Empagliflozin10mg | indrect | Low | Serious Imprecision |
| Canagliflozin300mg:Empagliflozin25mg | indrect | Low | Serious Imprecision |
| Canagliflozin300mg:Ertugliflozin15mg | indrect | Low | Serious Imprecision |
| Canagliflozin300mg:Ertugliflozin5mg | indrect | Low | Serious Imprecision |
| Dapagliflozin10mg:Empagliflozin10mg | indrect | Low | Serious Heterogeneity |
| Dapagliflozin10mg:Empagliflozin25mg | indrect | Moderate | Heterogeneity |
| Dapagliflozin10mg:Ertugliflozin15mg | indrect | Low | Serious Imprecision |
| Dapagliflozin10mg:Ertugliflozin5mg | indrect | Low | Serious Imprecision |
| Dapagliflozin5mg:Empagliflozin10mg | indrect | Low | Serious Imprecision |
| Dapagliflozin5mg:Empagliflozin25mg | indrect | Low | Serious Imprecision |
| Dapagliflozin5mg:Ertugliflozin15mg | indrect | Low | Serious Imprecision |
| Dapagliflozin5mg:Ertugliflozin5mg | indrect | Low | Serious Imprecision |
| Empagliflozin10mg:Ertugliflozin15mg | indrect | Low | Imprecision、Heterogeneity |
| Empagliflozin10mg:Ertugliflozin5mg | indrect | Low | Imprecision、Heterogeneity |
| Empagliflozin25mg:Ertugliflozin15mg | indrect | Low | Serious Heterogeneity |
| Empagliflozin25mg:Ertugliflozin5mg | indrect | Low | Serious Heterogeneity |
| **Urinary tract infection** | | | |
| Bexagliflozin20mg:PLACEBO | mixed | Moderate | Imprecision |
| Canagliflozin100mg:Canagliflozin300mg | mixed | Low | Serious Imprecision |
| Canagliflozin100mg:PLACEBO | mixed | Very low | Serious Imprecision、Incoherence |
| Canagliflozin300mg:PLACEBO | mixed | Moderate | Imprecision |
| Dapagliflozin10mg:Dapagliflozin5mg | mixed | Low | Serious Imprecision |
| Dapagliflozin10mg:PLACEBO | mixed | High |  |
| Dapagliflozin5mg:PLACEBO | mixed | Moderate | Imprecision |
| Empagliflozin10mg:Empagliflozin25mg | mixed | Moderate | Imprecision |
| Empagliflozin10mg:PLACEBO | mixed | Moderate | Imprecision |
| Empagliflozin25mg:PLACEBO | mixed | Moderate | Imprecision |
| Ertugliflozin15mg:Ertugliflozin5mg | mixed | High |  |
| Ertugliflozin15mg:PLACEBO | mixed | High |  |
| Ertugliflozin5mg:PLACEBO | mixed | High |  |
| Bexagliflozin20mg:Canagliflozin100mg | indrect | Low | Serious Imprecision |
| Bexagliflozin20mg:Canagliflozin300mg | indrect | Low | Serious Imprecision |
| Bexagliflozin20mg:Dapagliflozin10mg | indrect | High |  |
| Bexagliflozin20mg:Dapagliflozin5mg | indrect | High |  |
| Bexagliflozin20mg:Empagliflozin10mg | indrect | Moderate | Imprecision |
| Bexagliflozin20mg:Empagliflozin25mg | indrect | Low | Serious Imprecision |
| Bexagliflozin20mg:Ertugliflozin15mg | indrect | Low | Serious Imprecision |
| Bexagliflozin20mg:Ertugliflozin5mg | indrect | Moderate | Imprecision |
| Canagliflozin100mg:Dapagliflozin10mg | indrect | Moderate | Imprecision |
| Canagliflozin100mg:Dapagliflozin5mg | indrect | Moderate | Imprecision |
| Canagliflozin100mg:Empagliflozin10mg | indrect | Low | Serious Imprecision |
| Canagliflozin100mg:Empagliflozin25mg | indrect | Low | Serious Imprecision |
| Canagliflozin100mg:Ertugliflozin15mg | indrect | Low | Serious Imprecision |
| Canagliflozin100mg:Ertugliflozin5mg | indrect | Low | Serious Imprecision |
| Canagliflozin300mg:Dapagliflozin10mg | indrect | High |  |
| Canagliflozin300mg:Dapagliflozin5mg | indrect | Moderate | Imprecision |
| Canagliflozin300mg:Empagliflozin10mg | indrect | Moderate | Imprecision |
| Canagliflozin300mg:Empagliflozin25mg | indrect | Low | Serious Imprecision |
| Canagliflozin300mg:Ertugliflozin15mg | indrect | Low | Serious Imprecision |
| Canagliflozin300mg:Ertugliflozin5mg | indrect | Low | Serious Imprecision |
| Dapagliflozin10mg:Empagliflozin10mg | indrect | Low | Serious Imprecision |
| Dapagliflozin10mg:Empagliflozin25mg | indrect | High |  |
| Dapagliflozin10mg:Ertugliflozin15mg | indrect | High |  |
| Dapagliflozin10mg:Ertugliflozin5mg | indrect | High |  |
| Dapagliflozin5mg:Empagliflozin10mg | indrect | Low | Serious Imprecision |
| Dapagliflozin5mg:Empagliflozin25mg | indrect | Moderate | Imprecision |
| Dapagliflozin5mg:Ertugliflozin15mg | indrect | High |  |
| Dapagliflozin5mg:Ertugliflozin5mg | indrect | Moderate | Imprecision |
| Empagliflozin10mg:Ertugliflozin15mg | indrect | Moderate | Imprecision |
| Empagliflozin10mg:Ertugliflozin5mg | indrect | Moderate | Imprecision |
| Empagliflozin25mg:Ertugliflozin15mg | indrect | Low | Serious Imprecision |
| Empagliflozin25mg:Ertugliflozin5mg | indrect | Low | Serious Imprecision |
| **Genital mycotic infection** | | | |
| Bexagliflozin20mg:Placebo | mixed | Low | Serious Imprecision |
| Canagliflozin100mg:Canagliflozin300mg | mixed | Low | Imprecision、Heterogeneity |
| Canagliflozin100mg:Placebo | mixed | High |  |
| Canagliflozin300mg:Placebo | mixed | High |  |
| Dapagliflozin10mg:Dapagliflozin5mg | mixed | Low | Serious Imprecision |
| Dapagliflozin10mg:Placebo | mixed | High |  |
| Dapagliflozin5mg:Placebo | mixed | Low | Serious Imprecision |
| Empagliflozin10mg:Empagliflozin25mg | mixed | Very low | Serious Imprecision, Serious Incoherence |
| Empagliflozin10mg:Placebo | mixed | Low | Imprecision, Incoherence |
| Empagliflozin25mg:Placebo | mixed | Low | Imprecision、Heterogeneity |
| Ertugliflozin15mg:Ertugliflozin5mg | mixed | Low | Imprecision、Heterogeneity |
| Ertugliflozin15mg:Placebo | mixed | High |  |
| Ertugliflozin5mg:Placebo | mixed | High |  |
| Bexagliflozin20mg:Canagliflozin100mg | indrect | Low |  |
| Bexagliflozin20mg:Canagliflozin300mg | indrect | Low | Serious Imprecision |
| Bexagliflozin20mg:Dapagliflozin10mg | indrect | Low | Serious Imprecision |
| Bexagliflozin20mg:Dapagliflozin5mg | indrect | Low | Serious Imprecision |
| Bexagliflozin20mg:Empagliflozin10mg | indrect | Low | Serious Imprecision |
| Bexagliflozin20mg:Empagliflozin25mg | indrect | Low | Serious Imprecision |
| Bexagliflozin20mg:Ertugliflozin15mg | indrect | Low | Serious Imprecision |
| Bexagliflozin20mg:Ertugliflozin5mg | indrect | Low | Serious Imprecision |
| Canagliflozin100mg:Dapagliflozin10mg | indrect | Low | Serious Imprecision |
| Canagliflozin100mg:Dapagliflozin5mg | indrect | Low | Serious Imprecision |
| Canagliflozin100mg:Empagliflozin10mg | indrect | Low | Imprecision、Heterogeneity |
| Canagliflozin100mg:Empagliflozin25mg | indrect | Low | Imprecision、Heterogeneity |
| Canagliflozin100mg:Ertugliflozin15mg | indrect | Low | Serious Imprecision |
| Canagliflozin100mg:Ertugliflozin5mg | indrect | Low | Serious Imprecision |
| Canagliflozin300mg:Dapagliflozin10mg | indrect | Low | Serious Imprecision |
| Canagliflozin300mg:Dapagliflozin5mg | indrect | Low | Serious Imprecision |
| Canagliflozin300mg:Empagliflozin10mg | indrect | Low | Serious Imprecision |
| Canagliflozin300mg:Empagliflozin25mg | indrect | Low | Serious Imprecision |
| Canagliflozin300mg:Ertugliflozin15mg | indrect | Low | Serious Imprecision |
| Canagliflozin300mg:Ertugliflozin5mg | indrect | Low | Serious Imprecision |
| Dapagliflozin10mg:Empagliflozin10mg | indrect | Low | Serious Imprecision |
| Dapagliflozin10mg:Empagliflozin25mg | indrect | Low | Serious Imprecision |
| Dapagliflozin10mg:Ertugliflozin15mg | indrect | Low | Serious Imprecision |
| Dapagliflozin10mg:Ertugliflozin5mg | indrect | Low | Serious Imprecision |
| Dapagliflozin5mg:Empagliflozin10mg | indrect | Low | Serious Imprecision |
| Dapagliflozin5mg:Empagliflozin25mg | indrect | Low | Serious Imprecision |
| Dapagliflozin5mg:Ertugliflozin15mg | indrect | Low | Serious Imprecision |
| Dapagliflozin5mg:Ertugliflozin5mg | indrect | Low | Serious Imprecision |
| Empagliflozin10mg:Ertugliflozin15mg | indrect | Moderate | Heterogeneity |
| Empagliflozin10mg:Ertugliflozin5mg | indrect | Low | Imprecision、Heterogeneity |
| Empagliflozin25mg:Ertugliflozin15mg | indrect | Moderate | Heterogeneity |
| Empagliflozin25mg:Ertugliflozin5mg | indrect | Moderate | Imprecision |

# **Figure S8**: Confidence in evidence for all drugs compared to placebo

Confidence in the evidence for all SGLT-2 inhibitors versus placebo was assessed using the Confidence in Network Meta-analysis (CINeMA) framework, with seven outcomes included in the evaluation. Bar plots represent the percentage of outcomes corresponding to each evidence level: for example, 42.9% of reported outcomes for bexagliflozin 20 mg were rated high, 14.3% moderate, and 42.9% low. Treatments are ranked in descending order by the proportion of outcomes with high-certainty evidence—ertugliflozin 15 mg, for instance, had 71.4% of outcomes classified as high-level evidence.

# **Figure S9:Funnel plots of the efficacy and safety outcomes.**

A HbA1c B FPG


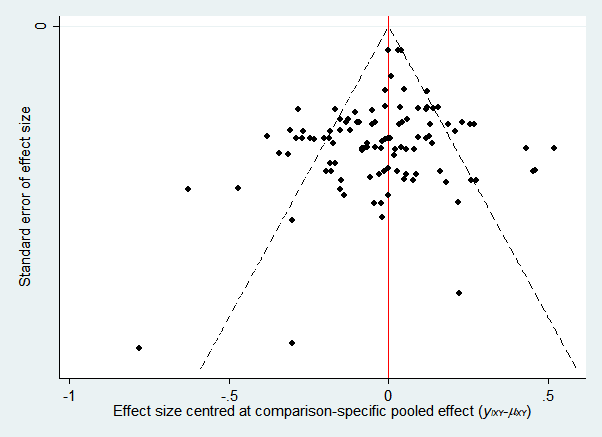

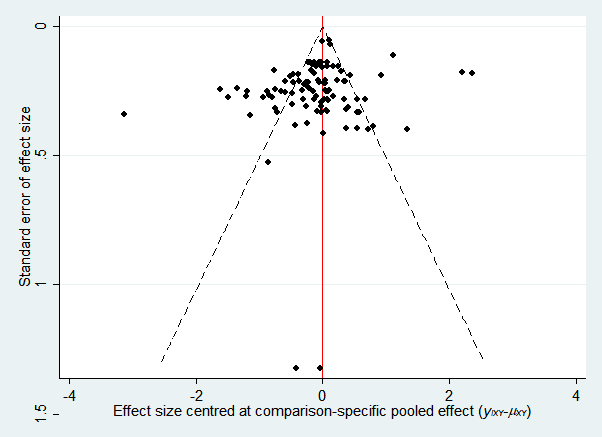


C Body weight D SBP


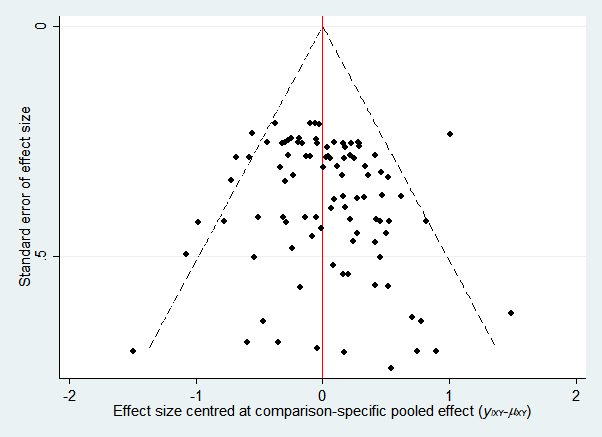

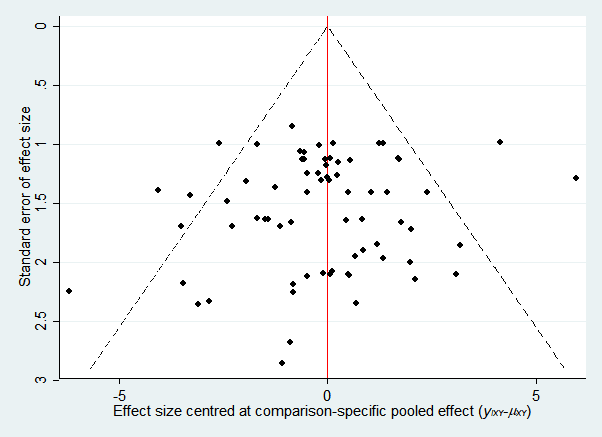


E DBP F hypoglycemia


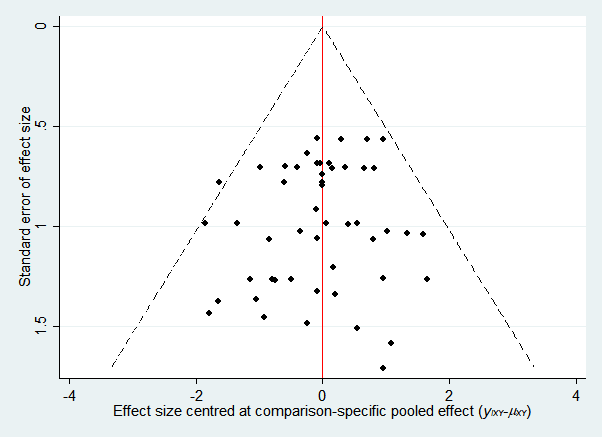

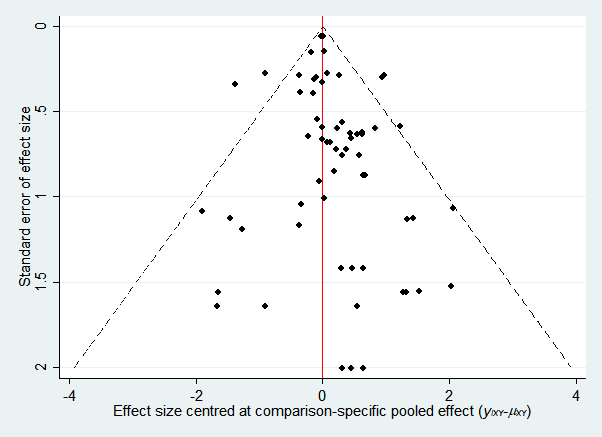


F Urinary tract infection H Genital mycotic infection.


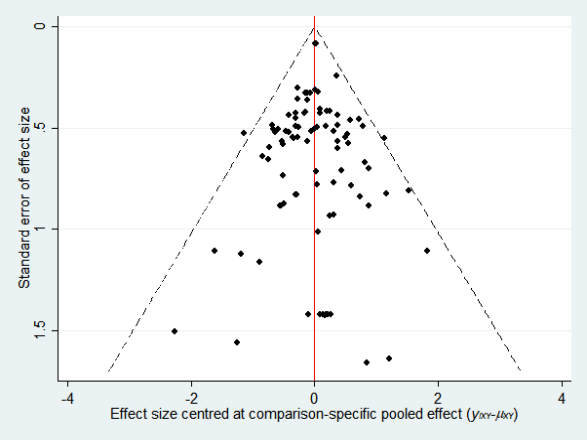

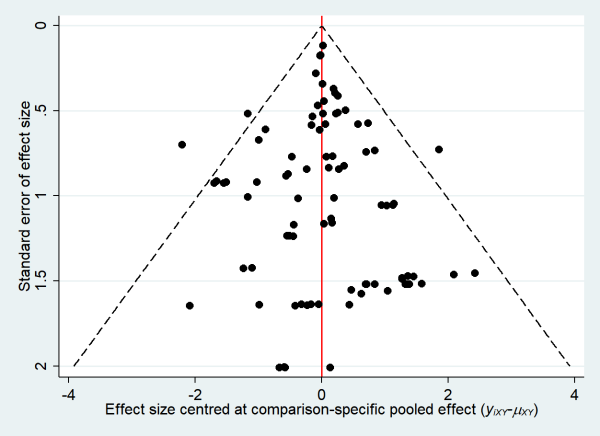


HbA1c: glycosylated haemoglobin; FPG: fasting plasma glucose; SBP: systolic blood pressure; DBP: diastolic blood pressure.

**Table S7 Results of Egger’s regression test**

| **Outcomes** | **p values** |
| --- | --- |
| HbA1c | **0.0143** |
| FPG | **0.0127** |
| Body weight | **0.126** |
| SBP | **0.0361** |
| DBP | **0.6956** |
| Hypoglycemia | **0.1264** |
| Urinary tract infection | **0.7607** |
| Genital mycotic infection | **0.8382** |

HbA1c: glycosylated haemoglobin; FPG: fasting plasma glucose; SBP: systolic blood pressure; DBP: diastolic blood pressure.

# **Figure S10: SUCRA of HbA1c excluding studies with contributes to heterogeneity**


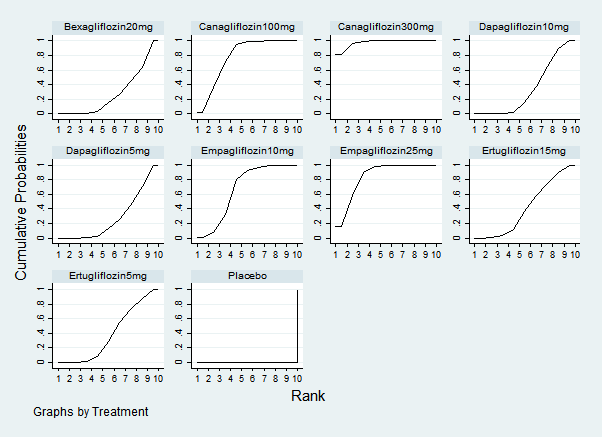


# **Figure S11: SUCRA of HbA1c excluding studies with small sample sizes**


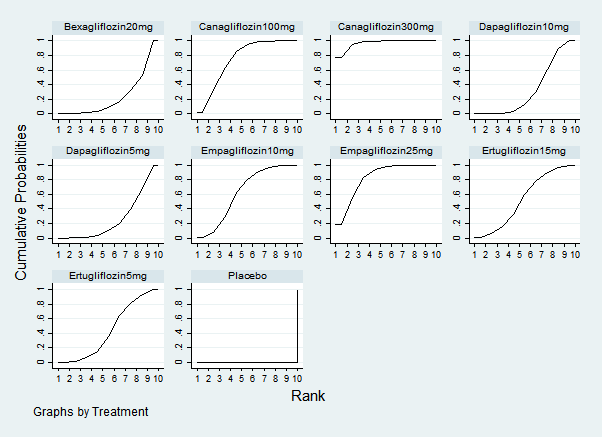

Supplement: Supplementary file 1 [file DataSheet1.docx]
